# State of the Science on Brain Insulin Resistance and Cognitive Decline Due to Alzheimer’s Disease

**DOI:** 10.14336/AD.2023.0814

**Published:** 2024-08-01

**Authors:** Elizabeth M Rhea, Manon Leclerc, Hussein N Yassine, Ana W Capuano, Han Tong, Vladislav A Petyuk, Shannon L Macauley, Xavier Fioramonti, Owen Carmichael, Frederic Calon, Zoe Arvanitakis

**Affiliations:** ^1^Geriatric Research Education and Clinical Center, Veterans Affairs Puget Sound Health Care System, Seattle, WA 98108, USA.; ^2^Department of Medicine, Division of Gerontology and Geriatric Medicine, University of Washington, Seattle, WA 98195, USA.; ^3^Faculty of Pharmacy, Laval University, Quebec, Quebec, Canada.; ^4^Neuroscience Axis, CHU de Québec Research Center - Laval University, Quebec, Quebec, Canada.; ^5^Departments of Neurology and Medicine, University of Southern California, Los Angeles, CA 90033, USA.; ^6^Rush Alzheimer’s Disease Center, Rush University Medical Center, Chicago, IL 60612, USA.; ^7^Biological Sciences Division, Pacific Northwest National Laboratory, Richland, WA 99352, USA.; ^8^Department of Physiology, University of Kentucky, Lexington, KY 40508, USA.; ^9^International Associated Laboratory OptiNutriBrain, Bordeaux, France and Quebec, Canada.; ^10^Univ. Bordeaux, INRAE, Bordeaux INP, NutriNeuro, UMR 1286, F-33000 Bordeaux, France.; ^11^Pennington Biomedical Research Center, Baton Rouge, LA 70808, USA.

**Keywords:** brain insulin resistance, Alzheimer’s disease, type-2 diabetes mellitus, insulin receptor, cognition

## Abstract

Type 2 diabetes mellitus (T2DM) is common and increasing in prevalence worldwide, with devastating public health consequences. While peripheral insulin resistance is a key feature of most forms of T2DM and has been investigated for over a century, research on brain insulin resistance (BIR) has more recently been developed, including in the context of T2DM and non-diabetes states. Recent data support the presence of BIR in the aging brain, even in non-diabetes states, and found that BIR may be a feature in Alzheimer’s disease (AD) and contributes to cognitive impairment. Further, therapies used to treat T2DM are now being investigated in the context of AD treatment and prevention, including insulin. In this review, we offer a definition of BIR, and present evidence for BIR in AD; we discuss the expression, function, and activation of the insulin receptor (INSR) in the brain; how BIR could develop; tools to study BIR; how BIR correlates with current AD hallmarks; and regional/cellular involvement of BIR. We close with a discussion on resilience to both BIR and AD, how current tools can be improved to better understand BIR, and future avenues for research. Overall, this review and position paper highlights BIR as a plausible therapeutic target for the prevention of cognitive decline and dementia due to AD.

## 1. Significance: The relation of diabetes, insulin resistance, and cognition

Type-2 diabetes mellitus (T2DM) and cognitive impairment related to Alzheimer’s disease (AD), are among the most prominent, fast-growing, and disabling chronic conditions of aging that negatively impact individuals and societies worldwide. The global prevalence of T2DM and AD were estimated to be 463 million and 57 million respectively in 2019 and are projected to increase to over 700 million and 150 million respectively in 2050 [1, 2]. Furthermore, T2DM has been shown to double the risk of all-cause dementia (clinical syndrome caused by a range of diseases), including dementia attributed to AD (a specific disease with characteristic biomarkers and pathologic features) and other causes [3, 4]. Notably, there are sex differences in T2DM related to cognitive impairment and the risk of developing AD, with women being more affected. Converging lines of evidence suggest that T2DM and dementia are closely associated in terms of risk factors and comorbidities. Several underlying mechanisms are postulated to link T2DM and dementia, and the shared etiology of these multifactorial diseases has been reviewed elsewhere [5]. Notably, T2DM is associated with cerebrovascular disease including stroke, which itself is a major cause of dementia (e.g., vascular contributions to cognitive impairment and dementia [VCID]) [6]. Additionally, inflammation, mitochondrial dysfunction, and other pathways are important features of insulin resistance and have been proposed as plausible links [7, 8]. Another key pathophysiological link is thought to relate to insulin resistance itself [9]. Defined as an impaired biological response of the body to insulin stimulation, peripheral insulin resistance has long been recognized to play a critical role in developing T2DM and in complications related to T2DM, including in the brain. Nevertheless, it was not until recent years that researchers found some aspects of insulin resistance in the brains of individuals with AD who did not have diabetes [10], supporting the idea that AD likely features metabolic disturbances that can present independently of T2DM, and involves insulin resistance in the brain. In addition, a large community-based prospective cohort study also shows that higher blood glucose levels and/or poorly controlled T2DM significantly increase the risk of dementia even in people without clinically-defined diabetes [11]. Moreover, in cognitively normal older adults, peripheral insulin resistance, indicated by high Homeostatic Model Assessment for Insulin Resistance (HOMA-IR) scores [12], has been associated with poorer performance on neurocognitive tests and abnormalities in biomarkers of AD, including increases in phosphorylated tau protein in the cerebrospinal fluid (CSF) and lower global cerebral glucose metabolism on positron emission tomography (PET) scans [13-16]. Given the close associations between T2DM, insulin resistance, dementia, and AD, researchers are currently examining these associations in a range of pre-clinical and clinical studies including testing the clinical benefit of anti-diabetic drugs such as metformin and insulin [17], as well as incretin analogs (e.g., glucagon-like peptide 1 [GLP-1] receptor agonists such as liraglutide, semaglutide) [18], in preventing and treating cognitive decline and dementia [19-23]. For the purpose of this work and future research, we define brain insulin resistance (BIR) as an inadequate response by cells located in the brain, including the cerebral vasculature, to secreted insulin: this inadequate response can be due to a limited CNS availability of insulin in its bioactive form, a limited expression of the insulin receptor (INSR) at the cell surface, a shift in INSR isoform expression, and/or diminished signaling events downstream from the INSR binding.

In the larger context of global health, the significance of the public health, challenge of both diabetes and dementia is further complicated by the Coronavirus Disease 2019 (COVID-19) pandemic caused by the virus SARS-CoV-2. On the one hand, both T2DM and dementia due to AD increase the risk of SARS-CoV-2 infection and the severity of COVID-19 [24, 25]. On the other hand, a diagnosis of COVID-19 has been associated with a higher risk for the development of both conditions, T2DM and dementia due to AD [26-28]. While there is much more research needed on the relation of COVID-19, these shared bi-directional associations further suggest a complex pathophysiology that is common to T2DM and dementia, and research is ongoing to better understand the long-term effects of COVID-19 on metabolism and brain function.

The focus of this review is to: 1) describe our understanding of insulin resistance in the brain and how it relates to, and differs from, peripheral insulin resistance, 2) describe in detail the contributors to BIR, including the role of the insulin receptor (INSR) particularly in the brain, 3) highlight current tools to study BIR, 5) elucidate how BIR may correlate with hallmarks of AD pathology underlying cognitive impairment and decline, and dementia, and 6) define where insulin resistance occurs within the brain and in individual cell types. We discuss pre-clinical, human post-mortem, imaging models in living humans, and human behavioral response to insulin research to interrogate this relationship. We close with a discussion about resilience both to AD and BIR, and ideas on how to improve our definition of BIR so that we may identify it earlier in the hopes of treating it and preventing cognitive decline and dementia.

## 2. Insulin in the brain

**Figure 1.**
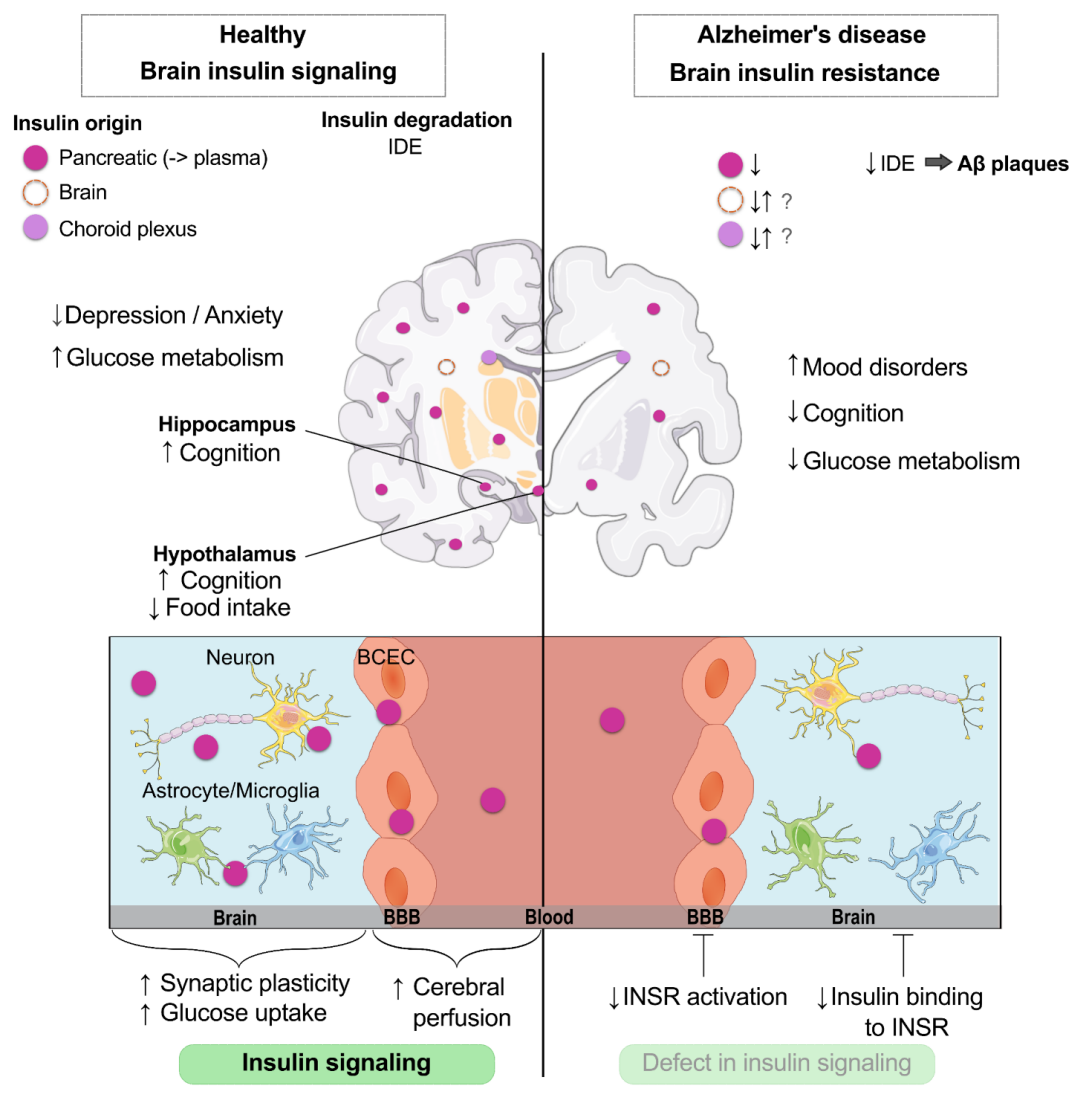
**Role of insulin in the healthy brain and impairments in brain insulin resistance (BIR) identified in Alzheimer’s disease**. Insulin in the brain predominantly originates from the pancreas, yet small amounts of insulin synthesis within the brain and choroid plexus have recently been discovered. Brain insulin is degraded by the insulin degrading enzyme (IDE) to regulate signaling. Under healthy conditions, brain insulin helps regulate mood, glucose metabolism, cognition, food intake, and brain perfusion. In Alzheimer’s disease, when BIR is present, there are decreased levels of brain insulin, decreased levels of IDE leading to increases in amyloid β plaques, increased mood disorders, worsened cognition, impaired glucose metabolism, and decreases in insulin receptor (INSR) activation. Parts of the figure were drawn by using pictures from Servier Medical Art.

Insulin was discovered in 1921 at the University of Toronto and is one of the most studied hormones over the last 100 years. Largely produced by the pancreatic islets β-cells, insulin is essential to life. Mechanisms of action of insulin include binding to the INSR to induce glucose uptake in many tissues, as well as promoting fatty acid and amino acid uptake. Insulin is utilized as a treatment for diabetes by millions of patients worldwide. In the brain, insulin has several recognized actions, most importantly on metabolism and supporting neuronal function like neuroplasticity, important for cognition [9, 29-31] and emotional behavior [32-34], by modulating brain networks [33, 35, 36] (Fig. 1). However, it should be noted that these roles for insulin in the brain may not extend to all regions. For example, insulin may impact memory directly in the hippocampus, but indirectly in the hypothalamus (i.e., through metabolic pathways). Insulin binds the INSR to induce a wide range of signaling events. Whether or not INSR activation induces changes in cellular glucose uptake in insulin-dependent glucose transporter (GLUT2/4) expressing neurons is still controversial. Reaching a better understanding of how the brain responds to insulin is likely to improve our capacity to possibly treat and prevent dementia, and possibly AD as well.

The brain is now recognized as an insulin-responsive organ [9, 29, 37] and a few researchers even postulate that there is a small degree of endogenous insulin production in the mammalian brain, including from the human choroid plexus [38, 39]. In the periphery, blood insulin levels increase postprandially, after being released by the pancreas. However, as opposed to peripheral organs, which have access to all insulin the pancreas can produce, the brain must predominantly rely on the blood-brain barrier (BBB) transport of insulin for access [40]. It remains largely unknown how postprandial changes in insulinemia affect the BBB and the brain. Whether insulin concentrations in the brain vary in the same way, given the limited BBB transport, is unlikely but remains unclear. Therefore, access of insulin to the central nervous system (CNS) interstitial fluid could be a regulatory mechanism, which upon failure, contributes to BIR. Even if brain insulin signaling is preserved, lower insulin levels could lead to a decreased response in this pathway. Additionally, the BBB has been shown to be a primary mediator of INSR signaling [41], which can impact insulin signaling in the whole brain [42]. The presence of the BBB contributes a unique source of enhanced regulation of brain insulin signaling. Whether BBB insulin resistance contributes to BIR or vice versa remains to be determined.

As we will discuss in detail in section 3, the degradation of insulin by the insulin-degrading enzyme (IDE) which can also regulate brain insulin availability, similar to what is observed in the periphery. Other events that contribute to BIR are changes in CNS cell type expression of the INSR, issues with the ligand-receptor interactions, and the inability to activate downstream signaling processes through receptor autophosphorylation [40].

While glucose is the preferred brain fuel substrate [43], brain glucose uptake is not regulated by physiological insulin levels in subjects with normal glucose processing. In healthy volunteers, hyperinsulinemia within the normal physiological range does not affect BBB glucose transport or net cerebral glucose metabolism [44, 45], as it is largely regulated by the insulin-independent GLUT1 and GLUT3 [46]. In contrast, insulin may stimulate greater glucose transport to the brain in insulin-resistant patients during hyperglycemia [47]. Additionally, the cognitive response to insulin has also been shown to be independent of glucose. That is, under euglycemic states, hyperinsulinemia appears to improve cognition in subjects with dementia attributed to AD [48]. While memory is also improved under hyperglycemic conditions, memory facilitation is greater with hyperinsulinemia. As an extensive discussion of glucose transport into and within cells of the CNS are beyond the scope of this review, we refer readers to a recent review on brain energy and neurodegeneration [49]. In the following section, we discuss in detail contributors to BIR which can include activation of the INSR and availability of insulin.

## 3. Contributors to BIR

### 3.1 Insulin receptor (INSR)

**Figure 2.**
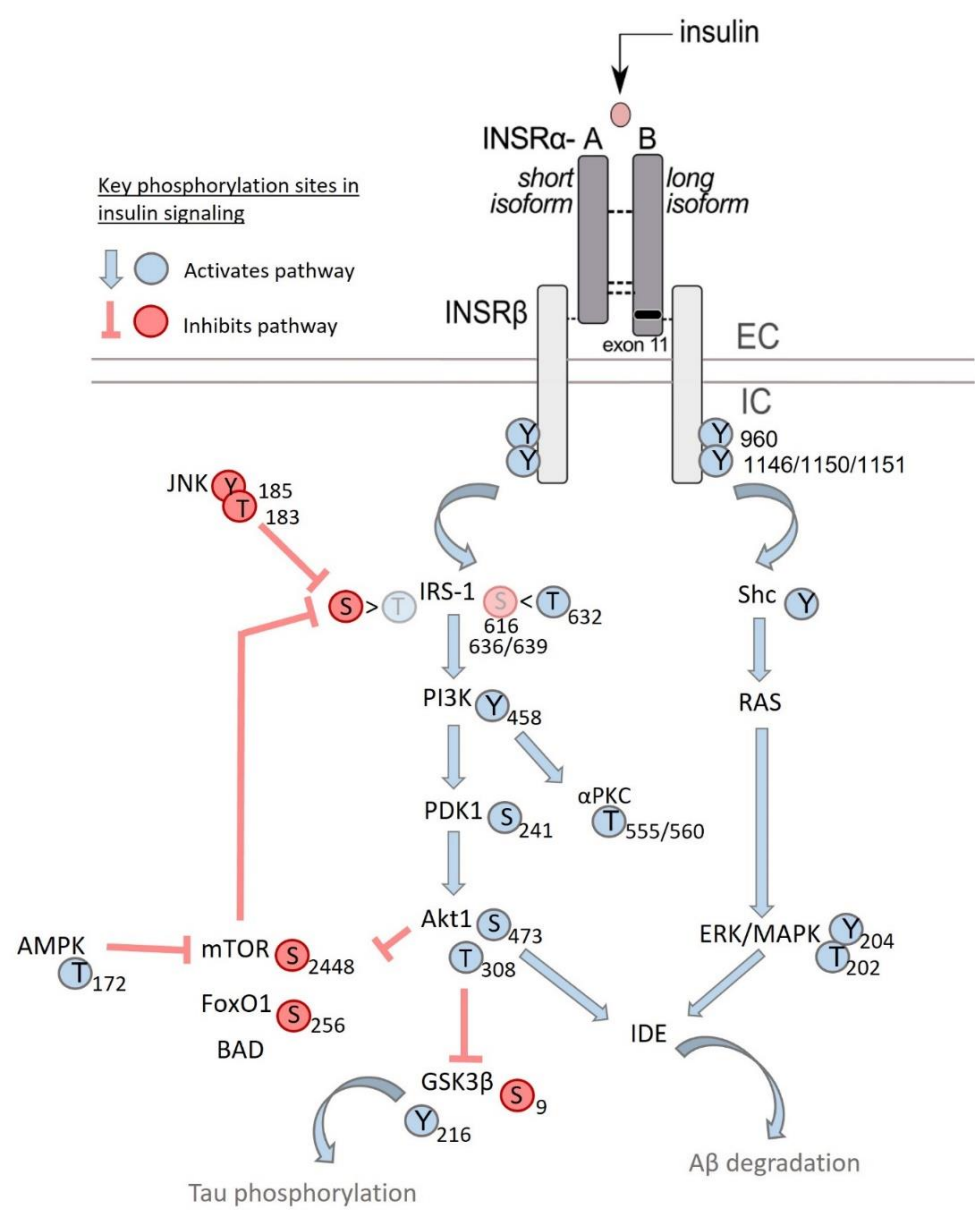
**INSR signaling pathway commonly investigated for BIR and phosphorylation sites interrogated**. Following insulin binding, the INSR is autophosphorylated at select tyrosine residues, resulting in activation. This triggers activation of the IRS-1 kinase, which can involve either activation on threonine residues or inhibition mediated by serine residues. Threonine phosphorylation of IRS-1 activates the downstream kinases PI3K, PDK1, and Akt1, which all are activated following phosphorylation. Akt activation inhibits GSK3β-mediated tau phosphorylation via tyrosine phosphorylation. Akt1 phosphorylation and subsequent activation can also inhibit mTOR inhibitory phosphorylation. IRS-1 activation can also be inhibited by JNK phosphorylation which limits threonine phosphorylation (activation) and enhances serine phosphorylation (inhibition). The non-canonical INSR signaling pathway involves Shc, Ras, and ERK/MAPK activation. Either Akt or the ERK/MAPK pathway can activate IDE which leads to Aβ degradation. In general, tyrosine and threonine phosphorylation of the kinases present in this pathway elicit activation of the kinase whereas serine phosphorylation inhibits activity of these kinases. Akt, protein kinase B; Aβ, β-amyloid peptide; BAD, Bcl-2-associated death promoter; EC, extracellular; ERK/MAPK, Mitogen-activated protein kinase kinase; FoXO, Forkhead; GSK3β, Glycogen synthase kinase-3 β; IC, intrecellular; INSR, insulin receptor; IRS1, insulin receptor substrates 1; JNK, c-Jun N-terminal kinases; mTor, mammalian target of rapamycin; PI3K, Phosphoinositide 3-kinases; αPKC, atypical protein kinase C.

**Figure 3.**
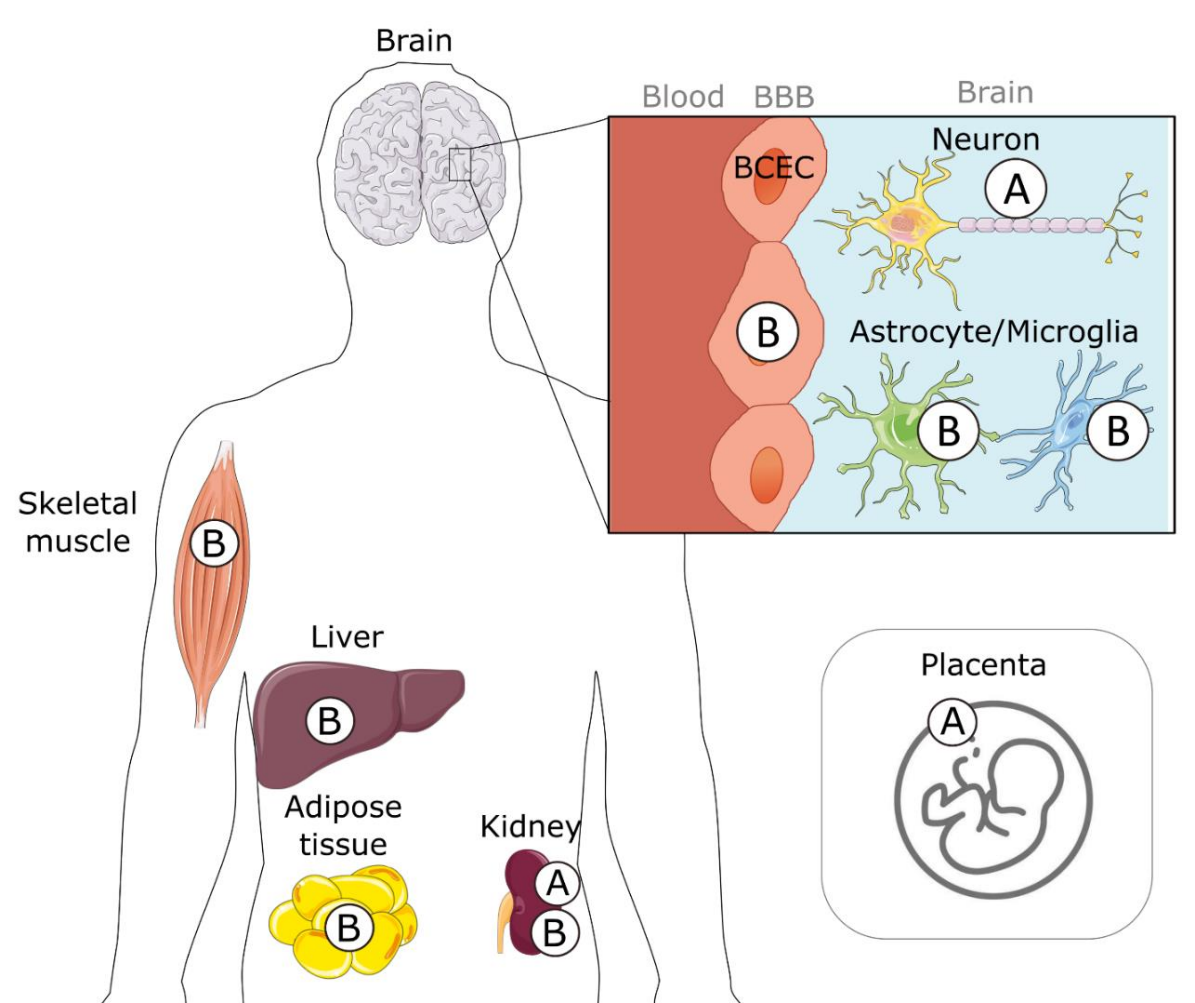
**Tissue specific expression of INSR isoforms**. Alternative splicing results in two isoforms of insulin binding INSRα: A and B. The expression of these isoforms varies between tissues, cell types, and even disease states. Parts of the figure were drawn by using pictures from Servier Medical Art. BBB: blood-brain barrier, BCEC: brain capillary endothelial cell.

The INSR is produced as a pro-receptor, undergoing proteolytic cleavage to become an active tyrosine kinase receptor. It is composed of two extracellular α chains (ligand binding) and two transmembrane β chains (intracellular signaling), as a homodimer (αβ)2 [50-52] (Fig. 2). During the maturation process, alternative splicing can produce two isoforms of the α-chain: the short A isoform (INSRα-A) truncated by 12 amino acids (exon 11) and the long B isoform (INSRα-B) [51, 53] (Fig. 3). Once insulin binds the extracellular α-chains, in a dose-dependent manner, a conformational change of the tetramer occurs and triggers intrinsic tyrosine phosphorylation within the intracellular β-chain [54-56]. This leads to binding to and phosphorylation of the insulin receptor substrate (IRS), which can ultimately trigger downstream intracellular signaling pathways such as RAS/MAPK (cell growth) and PI3K/Akt/mTOR (metabolism, cell growth), with particular relevance to AD [56-58] (Fig. 2). A decrease in INSR available at the cytoplasmic membrane [41] or a defective coupling to these downstream cellular cascades [10] are postulated as some of the causes of BIR, as well as AD.

### 3.1a Location

If BIR is predominantly reliant on the INSR, then it is important to define where the INSR is located in the brain. Autoradiography studies of ^125^I-insulin binding were one of the primary techniques used to assess the macroscopic localization of the INSR [59, 60]. In rat brain sections, the prominent binding levels of ^125^I-insulin are in the hippocampal formation [61], choroid plexus [62], hypothalamus [63] as well as the olfactory bulb, cortex, and cerebellum [64, 65]. One of the studies from that time used a cross-linkage strategy to covalently bind ^125^I-insulin to its receptor to show that most insulin binding sites were retrieved in cerebral microvessels in big animals (pigs and cows) [66].

Early protein analyses showed a wide INSRα distribution throughout the human brain with higher concentrations in the cortex than in the hippocampus and white matter [67]. Immunohistochemistry studies performed on paraformaldehyde-fixed brain sections reported localization of INSRβ in different areas of the rat forebrain (olfactory bulb, hypothalamus, and hippocampus), with staining in cells resembling neurons [68, 69]. The immunoreactivity of antibodies targeting the INSRα also suggested a neuronal localization in the precentral gyrus from human brain sections [67]. The differences between protein and ^125^I-insulin localization could be due to the specificity of the antibody and non-specific measurement of INSR localization since ^125^I-insulin binds other receptors.

More recent single-cell RNA transcriptomics analyses in mouse and human whole brain show that INSR mRNAs are largely found in endothelial and glial cells [70-73]. When microvessels are directly compared to parenchyma from the human and mouse brain, the INSR protein was found to be preferentially located on the cerebral vasculature constituting the BBB [41]. Such a high relative concentration of INSR in brain microvessels agrees with a growing number of studies showing that BBB INSR plays a key role in the action of insulin on the brain [41, 42, 74, 75]. The mismatch between cell type localization of INSR mRNA vs protein is likely due to the antibodies available for the INSR. Also, since mRNA levels do not always equate to protein levels, it will be important to pursue global brain proteomics not only on the regional localization of the INSR but also cell type expression.

### 3.1b Isoform expression

Further contributing to the complexity of INSR expression throughout the brain, is the question of whether the type of INSRα isoform expressed may also impact insulin signaling. While INSR is ubiquitously expressed, the ratio of INSRα-A/B changes throughout the body [76, 77] (Fig. 3). Insulin-responsive tissues such as the liver, skeletal muscle, adipose tissue, and kidney typically express high levels of INSRα-B [78, 79]. In cells from these organs, INSRα-A is highly expressed during fetal development with a shift toward the B isoform in adults [51, 80-83]. The relative protein abundance of INSR isoforms may result from differences in transcription and maturation processes [51]. The cancer field has provided a large variety of experimental settings to confirm the major impact of the INSRα-A/B ratio on insulin responsivity and downstream cellular response [51]. Not only does the INSRα-A/B ratio influence the relative affinity for ligands, such as insulin-like growth factor (IGF), proinsulin, and insulin, but it also impacts downstream signaling pathways [51, 81]. One specific mechanism is through the ability of (αβ)INSR to heterodimerize into hybrids with (αβ)IGF1-R, resulting in a difference in signaling [51, 56, 84-86]. Higher INSRα-A/B ratios have been observed in the liver of individuals with T2DM [87], as well as in adipocytes from patients with obesity [88] and can be reversed after weight loss induced by bariatric surgery [87, 88]. From this set of data, it has been postulated that a shift toward a higher INSRα-A/B ratio may disrupt the canonical insulin signaling pathway, leading to peripheral insulin resistance [51, 86, 89].

Changes in the INSRα-A/B ratio have recently been implicated in BIR as well. While INSR can be detected in most brain structures, the INSRα-A isoform is expressed chiefly by neurons, in contrast to the INSRα-B isoform which is primarily detected in endothelial cells, astrocytes, and microglia [51, 78, 90-93]. Consistently, higher levels of the B isoform are found in cerebrovascular extracts in both mouse and human brains, compared to parenchymal fractions [41].

In brains of individuals with AD, lower levels of the isoform INSRα-B are present in microvessels, resulting in an increase in the INSRα-A/B ratio [41]. In parenchymal fractions, only the A isoform was detectable, and it remained unchanged with AD pathology [41]. The mechanisms underlying this shift in ratio remain elusive but may involve preferential alternative splicing of INSR, microRNA regulation, and/or post-translational modifications [51]. Clinical relevance stems from the fact that insulin analogs can also be formulated to display higher specificities toward isoforms A and B of INSR [93, 94]. Further studies are needed to examine the relationship between BBB INSR isoform expression, BIR, and AD pathophysiology including amyloid β peptide (Aβ) clearance, Aβ/tau proteostasis, cerebrovascular pathology, and inflammation.

### 3.2 Availability of bioactive insulin

While changes in INSR or downstream pathways are most often evoked to explain insulin resistance at the molecular level [58, 95], it must also be kept in mind that the bioactivity of insulin itself may vary according to its quaternary structure or soluble component in the surrounding matrix. Human insulin is a classic amyloidogenic protein that aggregates at high temperatures in an acidic solution [96]. While endogenous aggregation of insulin released by the pancreas has not been observed, serum samples from Parkinson’s disease patients display an autoimmune response to insulin oligomers and fibrils, suggesting aggregated insulin may be present in this disease [97]. For example, insulin exists in hexamers or dimers, while only its monomer is expected to bind the INSR [50, 98-100]. The hexamer is the most common form, held together by two zinc ions, and can dissociate into the biologically active monomer for receptor binding. Insulin fibrillation from exogenous administration results in increasing insulin dosing due to a lack of functional insulin availability. The molecular features have been discussed in detail elsewhere [101, 102], and the interacting amino acids between insulin and the INSR have been mapped [99]. Stabilization of the hexameric, dimeric, or monomeric forms of insulin can prevent fibrillation and maintain biological activity.

Alternatively, insulin degradation by the IDE can impact the availability of insulin within the brain. Aβ is also a substrate of IDE [103, 104]. Brain protein expression of IDE is comparable to other major metabolic organs including the liver and pancreas [105]. The muscle is a tissue that expresses the highest amount of IDE protein, along with the stomach and small intestine. Most cell types within the CNS express *Ide* mRNA [106]. In particular, IDE is also detected at the BBB [41, 107, 108] which not only could be an additional regulator of how much insulin reaches the brain but also a way to control BBB INSR signaling.

As mentioned above, the BBB is also a critical regulator of brain insulin availability [30, 41, 109]. BBB insulin transport is altered under many different pathological conditions, including AD and T2DM [60, 109]. We will discuss later the involvement of the BBB in regulating brain insulin levels and how changes in the transport of insulin into the brain may impact BIR. Now that we have a clearer definition of BIR and how it may arise, we will next discuss ways to interrogate BIR in animal models and humans.

## 4. Tools to Study BIR

**Figure 4.**
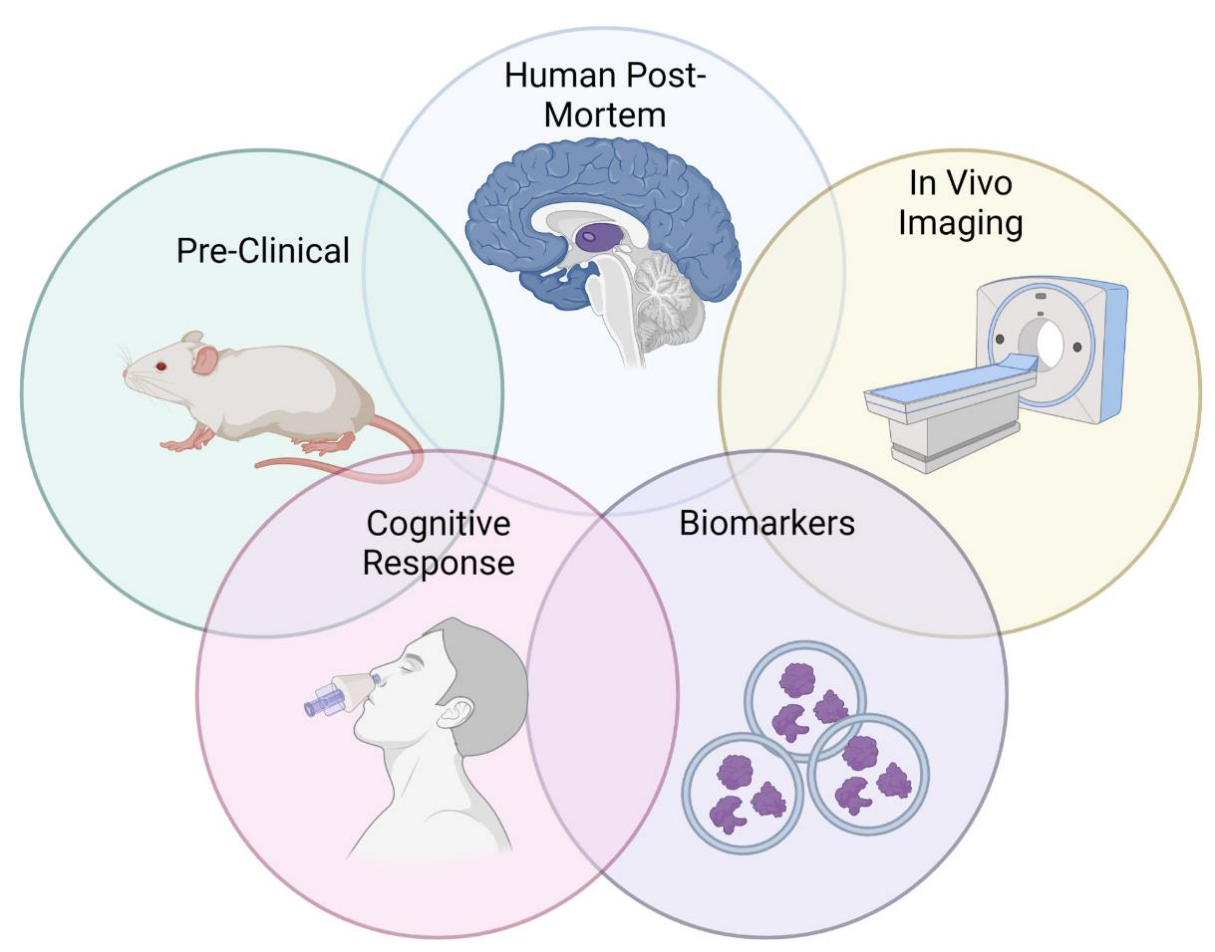
**Current tools commonly used to interrogate BIR**. We highlight data that utilizes the following tools to investigate BIR: pre-clinical rodent models, human post-mortem in situ insulin stimulation, in vivo neuroimaging especially following insulin stimulation, biomarkers including CNS-derived exosomes, and cognitive response to insulin treatment. Figure created using Biorender.

BIR can be interrogated in various ways in mammals from rodents to humans (Fig. 4). Changes in protein levels of the INSR signaling pathway are some of the first endpoints most commonly used to define BIR. Phosphorylation of INSR, of its partner IRS and/or of downstream players such as serine/threonine kinase Akt, glycogen synthase kinase 3 (GSK3), and mTOR [56] are some of the primary mediators (Fig. 2). In most instances, higher tyrosine phosphorylation is a sign of activation whereas serine/threonine phosphorylation is a sign of less active INSR and IRS proteins [56, 58].

The most common and reliable technique to measure activation of the signaling cascade remains Western immunoblots as they readily allow the parallel measurement of phosphorylation status in relation to the total number of proteins in the same sample. However, Western immunoblots are semi-quantitative and require a relatively high number of samples. Enzyme-linked immunosorbent assays (ELISAs) are more quantitative and high-throughput but have difficulties detecting phosphorylation sites due to inadequate antibodies. Phosphoproteomic analyses utilizing mass spectrometry have demonstrated tyrosine phosphorylation of a dozen of proteins in response to insulin [110, 111]. While these techniques are promising in uncovering novel mechanisms, they are currently costly and may be less applicable for studies involving several samples, as subsequent confirmation with Western immunoblots or ELISAs is often still necessary. However, just as bulk RNA sequencing has become a popular, genomic tool that has become more affordable over the last decade since it was developed, it is likely phosphoproteomic technology will continue to develop as well [112]. In addition, to obtain further insights on mechanisms, recent techniques utilize human post-mortem tissue, a limited and precious resource to study brain insulin signaling, employ immunohistochemistry and ex vivo stimulation of post-mortem brain tissue with insulin [10]. The electrophysiological response to insulin in rodents or post-mortem tissue can also be used to identify the modulation of neuronal activity [33].

These changes in the INSR signaling pathway can be assessed not only in various rodent models of BIR, including diet-induced, toxin, transgenic, and non-transgenic models but also in human post-mortem tissue. Yet, these latter tissues are not commonly available and methods to collect tissue affect measurement (e.g., need a short postmortem interval to be able to measure response to insulin stimulation). Other tools that are more commonly used as read-outs of BIR in living subjects are neuroimaging and biomarkers (see below).

### 4.1 BIR assessment in rodent models

Rodent models remain an essential tool for studying insulin response in vivo and inform human research, as well as allow testing hypotheses in experiments based on human data, including common pre-disposing genetic and lifestyle risk factors of BIR and AD like the apolipoprotein E genotype, a high fat diet, and decreased physical activity. In the last few decades, significant efforts have been invested in defining the molecular signature of INSR activation in the brain of rodents. While these cascades have been delineated mostly in various cultured cell models [56, 58], they can be replicated in the muscle and the liver following insulin injection [113-119]. A reduction of this response, assessed with these methods, is then interpreted as evidence of insulin resistance.

**Table 1:** Peripheral and central insulin administration in animal models.

	Ref.	Mouse models	Insulin administration		Brain zone studied (Method)	Insulin administration effects:		
			Insulin type, vehicle	fasting information, delay, and death		INSR and IRS-1 activation	Signaling downstream kinases	Others protein activation/phosphorylation
**Peripheral insulin administration**	**Intraperitoneal injection**							
	[119]	5 to 6-month-old C57Bl/6 male	vehicle or insulin (5 IU/kg) *no insulin details*	2h fasting Injection 0 to 60 min before death (cervical dislocation)	Left Cx (WB)		Moderate ↓ of pPI3K(Y^458^), pAkt(S^473^), pGSK3β(S^9^) at 15 min, followed by ↑ until 60 min ↓ pmTor(S^2448^) (15 min), = pAMPK(T^172^) and↓ pErk(T^202^/Y^204^) (with time 0 -> 60min)	↓ pTau(S^199^), (T^205^), (T^212^), (S^214^), (T^217^), (S^262^), (S^396^), and (S^404^) at 15 min and ↑ after 30 min
	[116]	4 to 6-month-old 1) male C57Bl/6 ob/ob (OB), lean ob+ (CON) or 2) male, female C57Bl/6 +/- HFD (HFF)	vehicle or insulin (1 IU/kg) *no insulin details*	*No fasting indication* Injection 15min before death (intracardiac perfusion)	Whole hemisphere (WB)		CON: ↑ pAkt(S^473^), pGSK3β(S^9^), pmTor(S^2448^) OB or HFF: = pAkt(S^473^), pGSK3β(S^9^), pmTor(S^2448^) (but > CON saline)	CON: ↑ pαPKC(T^555/560^), pFoxO1(S^256^) and pFoxO3a(S^253^) OB or HFF: = pαPKC(T^555/560^), pFoxO1(S^256^) and pFoxO3a(S^253^) (but > CON saline)
	[118]	6-month-old inducible liver-specific insulin receptor KO (iLIRKO) 50 or 100% INSR deletion	vehicle or insulin (1 IU/kg) human insulin	Non-fasted Injection 10 min before death	Whole Brain (WB)	= INSRβ	50% deletion: ↓ pAkt(S^473^), pErk(T^202^/Y^204^), pp70S6K(T^421^/S^424^) 100% deletion: ø pAkt(S^473^), pErk(T^202^/Y^204^), pp70S6K(T^421^/S^424^)	
	[342]	8 to 12-week-old C57BL/6 male mice	saline or insulin (300 IU/kg) bovine pancreas insulin	Non-fasted Injection 2.5h before death (cervical dislocation, no anesthesia/perfusion)	Hpc and neocortex (WB)		↑ pGSK3β(S^9^)/GSK3β, pJNK(Y^185^)/JNK = pGSK3β(Y^216^)/GSK3β, pErk(T^202^/Y^204^)/Erk	
	[129]	6-7-week-old male C57Bl/6 STZ-ip	PBS or insulin (5 IU/kg) bovine pancreas insulin	o/n fasting Injection 5 to 30 min before death (decapitation)	Hpc and Cx lysates (WB)		↑ rapid, transient pAkt (T^308^, S^473^), pGSK3β(S^9^), pGSK3α(S^21^) (peak at 5 min) = total Akt, GSK3β and GSK3α	
		**6-7 week-old male C57Bl/6 STZ-ip 3 days before ins**			Hpc and Cx lysates (WB)		↓ pAkt (T^308^, S^473^) ↑ pGSK3β(S^9^), pGSK3α(S^21^) (baseline is high) = total Akt, GSK3β or GSK3α	
	**Intravenous injection**							
	[117]	12-month-old APOE3 and APOE4 mice	saline or insulin (33.8 IU/kg) human insulin	6h fasting Tail vein injection 5 min before death (intracardiac perfusion)	Microvessels (WB)	= INSRβ, IRS1		↓ RAGE, = LRP1 (APOE4 and E3)
					PTCx TBS-soluble (WB)		↑ pAkt(S^473^)/Akt (APOE4 > E3) ↑ pGSK3β(S^9^), pErk(T^202^/Y^204^) (APOE4 and E3) = pPDK1(S^241^)/PDK1, pPI3K(Y^458^), pERK(T^202^/Y^204^), pJNK(T^183^/Y^185^)/JNK, pmTor(S^2448^) (APOE4 and E3)	↑ pTau(S^202^) (APOE4 > E3) ↑ pTau (S^396/404^, T^181^) = pTau (T^231^)
								= RAGE, LRP1 (APOE4 and E3)
	[120]	C57Bl/6 male young (10-12 weeks) and aged (77-95 weeks)	saline or insulin (1 IU/mouse for 10min) human insulin	o/n fasting Inferior vena cava injection for 10min (decapitation)	Whole brain lysate (WB)		↑ pAkt(S^473^)/Akt in young mice only = pAkt(S^473^)/Akt in aged mice	
	[115]	15-month-old female 3xTg-AD +/- HFD	saline or insulin (33.8 IU/kg) human insulin	6h fasting Tail vein injection 5 min before death (intracardiac perfusion	PTCx soluble fraction (WB)		↑ pAkt(S^473^) = pGSK3β(S^9^) (no diet effect)	= pTau (S^202^, T^181^, S^396^)/Tau (also in Hpc)
								= RAGE, LRP1, IDE (soluble and membrane)
	[121]	8-week-old C57Bl/6	saline or insulin (1 mIU or 4 IU) Rapid human recombinant insulin (Actrapid®)	8h fasting Inferior vena cava injection 5 to 20 min before death (intracardiac perfusion)	Whole brain (WB)	↑ pINSR from 5 to 20 min (1 mU) with immunoprecipitated INSR	↑ pAkt(S^473^)/Akt (max 10 min), ↑ pGSK3β(S^9^)/GSK3β with time (max 10 to 20 min), ↑ pERK/ERK (max 10 min) (1 mU)	↑ pTau(S^202^) since 5 to 20 min (4 U > 1 mU) = pTau(T^231^) (4 U and 1 mU)
	[122]	8-week-old male mice Akt2^-/-^, Akt3^-/-^ (DKO)	saline or insulin (10 mIU/g) human recombinant insulin	o/n fasting Inferior vena cava injection 12 min before death	Whole brain (WB)		= pAkt(S^473^), pGSK3β(S^9^)	
	**Hyperinsulinemic clamps**							
	[123]	3- or 12-month-old heterozygous APP/PS1	0.1%BSA-PBS, or insulin (4 mIU/kg/min) regular human recombinant insulin (Humulin®R)	*No fasting indication* Hyperinsulinemic-euglycemic clamp 60 or 90 min before death	Hpc and Hyp lysates (ELISA)		= pAkt(S^473^)/Akt (young and aged APP/PS1)	
	[121]	12-week-old C57Bl/6 male or NIRKO mice	saline + 0.1%BSA, or insulin (200 mIU/kg) regular human insulin (Actrapid®)	o/n fasting Right internal jugular vein hyperinsulinemic-euglycemic clamp 10 to 60 min before death	Whole brain (WB)		↑ pErk/Erk (max 10 min) NIRKO: = pAkt(S^473^)/Akt, pGSK3β(S^9^)/GSK3β, pErk/Erk (no variation)	↑ pTau(S^202^) since 10 to 60min NIRKO: = pTau(S^202^) from 0 to 20 min (no variation)
**Central insulin administration**	**Intracerebroventricular injection**							
	[125]	8-day-old male layer chicks	saline with 0.1% Evans blue and 43 µM chloric acid, or insulin (100 pmol/chicks) porcine insulin	3h fasting 9 days injections, death 30 min after the last one (decapitation)	Medulla (WB)		↑↑ pAkt/Akt, ↑ pErk/Erk	
	[124]	young (4 months) and aged (24 months) male Wistar rats	saline or insulin (1 or 20 mIU) regular human recombinant insulin (Humulin®R)	*No fasting indication* 5 days injections, death after the last one	Right Hpc (WB)	Young rats: = INSRβ Aged rats: ↑ INSRβ	Young rats: ↑ pGSK3β(S^9^)/GSK3β, = mTor Aged rats: ↑pGSK3β(S^9^)/GSK3β, ↑ mTor	
	[120]	young (10-12 weeks) and aged (77-95 weeks) C57Bl/6 male mice	saline or insulin (3.75 mIU/5µl 5.41nmol/ml) human insulin (Actrapid®)	o/n fasting	Whole brain lysate (WB)		↑ pAkt(S^473^)/Akt in young and aged mice	
	[126]	2-month-old CF1 (wild-type) mice	saline or insulin (5mIU) *no insulin details*	Non-fasted Injection 15min or 24h before death (decapitation)	Hpc synaptic membrane (WB)	↑ INSR (15 min > 24h)		
					Hpc homogenate (WB)	= INSR ↑ pINSR(Y) (5 mUI 15 min > 24h)	↑ pAkt(S^473^) (15 min)	
	[128]	adult male Sprague-Dawley rats	saline or insulin (6 mU/6µl) human recombinant insulin	o/n fasting Injection 15min or up to 60 min before death	Hpc (WB)	= INSRβ	↑ pAkt/Akt with time between 15-45min, and ↓ after 60 min	
	**Intracarotid perfusion**							
	[343]	16-month-old Non-transgenic (NTg) or 3xTg-AD (3x)	oxygenated bicarbonate buffered + saline or insulin (350 nM) regular human recombinant insulin (Novolin®ge Toronto)	Non-fasted Intracarotid perfusion 2 min before death (decapitation)	Microvessels (WB)	NTg; ↑ pINSRβ(Y^1150/1551^)/INSRβ*, = pro-INSR, INSRβ, INSRα 3x: = pINSRβ(Y^1150/1551^)/INSRβ*, = pro-INSR, INSRβ, INSRα		= BACE1, eNOS, caveolin-1, P-gp, LPR1, RAGE, Neprilysin, IDE (NTg and 3x)
	**Intracerebral injection**							
	[123]	3- or 12-month-old heterozygous APP/PS1	artificial CSF +/- insulin (40 or 400nM for 1h) regular human recombinant insulin (Humulin®R)	*No fasting indication* Hippocampal reverse microdialysis 1h before death	Hpc around the injection site (ELISA and WB)		↑ pAkt(S^473^)/Akt (400 nM) (young and aged APP/PS1)	
	[127]	1-month-old male Sprague-Dawley rats	artificial ECF +/- insulin (100µU) regular human recombinant insulin (Humulin®R)	*No fasting indication* Hippocampal reverse microdialysis 10 min before death	Hpc around the injection site (WB)		↑ pAkt	

Summary of insulin delivery interventions (peripheral or central) in animal models and their impact on downstream activation of insulin signaling. The majority of the studies have investigated downstream signaling rather than insulin receptor (INSR) and first effector (IRS1) activation. Studies include control animals as well diet-induced metabolic impairment (HFD), type 2 diabetes mellitus (T2DM), or genetic insulin resistance models (LIRKO, ob/ob, STZ-ip), insulin signaling knock-out model (DKO, NIRKO), or in Alzheimer’s disease neuropathological models (APP/PS1, 3xTg-AD). ↑, ↓ or = indicate increased, decreased or equal levels, respectively, following insulin stimulation compared to control. *Abbreviations : AD, Alzheimer’s disease; Akt, Protein kinase B; AMPK, AMP-activated protein kinase; BACE1, β-site APP cleaving enzyme 1; BSA, bovine serum albumin; Cx, cortex; ELISA, enzyme-linked immunosorbent assay; ECF, extracellular fluid; eNOS, Endothelial NOS or nitric oxide synthase; Erk, Extracellular signal-regulated kinase; FoxO, forkhead box transcription factors; GSK3β, Glycogen synthase kinase-3β; HFD, high fat diet; Hpc, hippocampus; Hyp, hypothalamus; IB, immunoblot; IDE, Insulin-degrading enzyme; INSR, Insulin receptor; IP, immunoprecipitated; IRS1, insulin receptor substrate 1; IU, international unit; JNK, c-Jun N-terminal kinases; KO, knock-out; LRP1, Low density lipoprotein receptor-related protein 1; mTor, mammalian target of rapamycin; ob/ob, obese mice leptin resistant; P70S6K, Ribosomal protein S6 kinase β-1; PDK, phosphoinositide-dependent protein kinase-1; P-gp, p-glycoprotein; PI3K, Phosphoinositide 3-kinase; PTCx, parieto-temporal cortex; RAGE, Receptor for advanced glycation endproducts; STZ-ip, Streptozotocin-intraperitoneally injection; WB, western blotting; αPKC, protein kinase Cα.*

However, generating clear proof of INSR activation in the brain triggered by a rise in insulin in the blood circulation has proven challenging, particularly when a small number of animals are used. Table 1 summarizes the range of studies published. From this, we can see that the modes of administration used are varied, associated with different pre-clinical paradigms, brain regions investigated, type or dosage of insulin, dietary status, and euthanasia (method and delay). A first observation is that very few studies directly addressed INSR or IRS-1 phosphorylation, likely due to limited reliable antibodies, and most assessed relative phosphorylation of Akt, GSK3β, or Erk1/2 (Table 1). Intravenously injected insulin at supraphysiological doses is reported to increase pAkt(S^473^)/Akt ratios in the brain in several but not all studies [115, 117, 120-122]. However, when a hyperinsulinemic clamp is used to fully control blood insulin concentrations, no increases in interstitial fluid levels of insulin or activation of insulin signaling is detected in the hippocampus, contrasting with a clear rise in pAkt in the muscle [123]. Alterations of other kinases are harder to ascertain, and any rise in pAkt(S^473^)/Akt ratio detected in the brain remains very small compared to the response triggered in the muscle of the same animals [115, 117, 123] (Table 1 Peripheral). By contrast, studies using direct intracerebral or intracerebroventricular (ICV) infusion of insulin consistently show increased activation, mediated by phosphorylation of Akt and GSK3β close to the injection site in the whole brain, hippocampus, or medulla [120, 123-128] (Table 1 ICV). The activation of the cellular cascade downstream of INSR is fast. When detected, the phosphorylation of Akt occurs within 15 minutes after stimulation by insulin in vivo and in vitro [56, 119, 121, 126-129] (Table 1). AD-related factors such as older age or the presence of apolipoprotein E4 (ApoE4) have been found to result in a diminished [120] or enhanced [117] insulin-induced phosphorylation of Akt, respectively (Table 1 Peripheral).

Although less commonly measured, the phosphorylation of INSRβ is the initial activation step specifically following insulin or IGF binding [56]. A more limited number of publications report activation of INSRβ following insulin administration through intravenous, ICV, or intracarotid routes [41, 121, 126] (Table 1). For example, INSRβ phosphorylation can be more readily observed in microvessel extracts after intracarotid administration [41], suggesting a response preferentially located in the endothelium of the brain vasculature.

Within neurons, one major consequence of signaling cascades downstream of the INSR is the modulation of ionic channels which promotes changes in electrical activity. The fact that insulin is able to modulate the excitability or firing rates of neurons has been determined in several brain regions including the hypothalamus, ventral tegmental area (VTA), hippocampus, and dorsal raphe nucleus using patch-clamp electrophysiology. Interestingly, these regions are brain areas involved in functions including feeding behaviors, memory, emotion, sleep, or motivation; functions which are also modulated in response to intranasal delivery of insulin. Thus, in the hypothalamus, insulin has been shown to modulate the electrical activity of the anorexigenic pro-opiomelanocortin neurons [130-133], the orexigenic neuropeptide Y neurons [134, 135], neurons within the ventromedial hypothalamus involved in the control of the counter-regulatory response to hypoglycemia [136], or melanin-concentrating hormone neurons of the lateral hypothalamus [137]. In extra-hypothalamic regions, insulin has been shown to modulate the electrical activity of VTA dopaminergic neurons [138, 139], hippocampus neurons [140], serotonergic neurons [33], or olfactory neurons of the olfactory epithelium [141]. To our knowledge, only two families of ionic channels have been suggested to mediate the effect of insulin including potassium ATP-dependent channel responsible for the hyperpolarizing effects of insulin [134-136, 142], or canonical transient-receptor potential (TRPC) involved in the excitatory effect of insulin [130, 132]. Interestingly, in a mouse model of T2DM, the response to insulin is impaired in several of these neuronal populations [33, 139, 142] supporting evidence for BIR. Of note, in these patch-clamp studies, the direct effect of insulin on neurons suggests BIR is present in the neuron itself. These data are particularly important to aid in differentiating between BIR at the neuronal level versus limited BBB transport of insulin under various conditions in vivo studies.

In sum, these studies show that the detection of INSR activation by circulating insulin is difficult, but perhaps clearer when insulin is injected directly into the brain circumventing BBB transport or when cell populations are independently assessed (i.e., microvessels following carotid insulin injection or neuronal patch clamp studies). Supraphysiological doses or dietary modifications [143] are also often necessary to increase brain levels of insulin. However, supraphysiological doses may not accurately reflect what occurs under normal conditions. First, insulin BBB transport is saturable [144, 145]. Therefore, increasing doses of insulin above the saturable rate will not result in further increases in brain insulin. Second, the activation of Akt/GSK3β/Erk pathway is more frequently reported than the direct assessment of the phosphorylation of INSR/IRS-1. This issue limits data interpretation because while we can reasonably attribute a higher phosphorylation rate of INSR and IRS to the action of insulin, downstream kinases involved in the INSR cascade are shared by a wide variety of cell signaling pathways [146], which makes it more difficult to tightly link such endpoints to a specific response to insulin. Finally, studies are most often performed on brain homogenates, which do not provide cellular localization and may dilute the signal of INSR-activated cells, as will be discussed later. Keeping in mind these challenges, pre-clinical models offer suitable means to investigate the mechanism underlying BIR in vivo. Since blood-derived insulin must interact with the BBB before it can access downstream cell cascades in the CNS, studying brain insulin response within the neurovascular unit may also facilitate the investigation of BIR.

### 4.1a Diet-induced models

The most frequently used method to generate insulin resistance in an animal is to induce T2DM following an excessive intake of fat and/or sugar [33, 115, 143, 147-149]. The scientific literature is replete with demonstrations of acquired signs of insulin resistance in the periphery, most particularly in the liver or muscle [113-115, 150].

Combining transgenic induction of AD-like pathology with a high-fat diet (HFD) has been useful to shed light on AD-relevant brain-periphery interactions. Spontaneous changes in glucose response are often described in these models and are greater with aging [123, 143, 151-155] (Fig. 2). Furthermore, in rodent models of AD neuropathology, the intake of a HFD generally aggravates cognitive performance and brain Aβ pathology, while effects on tau and synaptic markers, including synaptophysin and syntaxin-3, are less consistent [115, 149, 153, 156-163]. However, an alternative study has shown transcriptional levels of the INSR in the hippocampus are decreased in an AD mouse model (Tg2576) and feeding a HFD for 10 months to these mice restored levels to controls [164]. Cognition was also improved in the AD HFD mice. Sex-dependent peripheral glucose intolerance and insulin resistance are typically observed as well, with females often affected earlier [157, 160, 165]. A single insulin injection led to a restoration of soluble Aβ levels in cortex and memory function in the 3xTg-AD mouse model on a HFD, possibly associated with greater Aβ clearance through the BBB [115, 166]. How insulin and BIR is able to alter Aβ levels is largely unknown, but it is thought that brain insulin can impact Aβ clearance [167] and/or processing [168].

While a HFD paradigm is efficient to generate insulin resistance in the periphery, signs of BIR have been less studied so far. One of the first studies using HFD-fed Tg2576 mice reported a decrease in basal INSR activation and downstream signaling (see Table 1) in the cortex, compared to a chow-fed control group [169]. AD mice on a HFD had poorer memory, increased brain Aβ neuropathology with higher levels of Aβ_40/42_ in the hippocampus, an increased Aβ production in the cortex (more APP γ-C-terminal fragments and plaque burden), and lower levels of IDE [169]. This was mirrored in a similar study exploring HFD in another AD mouse model (A7-Tg), where HFD increased both soluble Aβ_42_, insoluble Aβ_40_ and Aβ_42_, and amyloid plaque formation, which was due to altered ISF Aβ_42_ clearance [143]. In another study, no difference in basal INSRβ activation was observed in the hippocampus of wild-type (WT) or the APP/PS1 mouse model of AD after 6 months of HFD, only a decrease in pAkt(S^473^), along with, surprisingly, higher relative phosphorylation of other kinases (Table 1) [170]. In addition, 3-month-old HFD-fed APP/PS1 showed no differences in the activation of these kinases in the hippocampus and no change in tau protein phosphorylation of various epitopes [171]. One study reported HFD accelerates the ApoE4 impairment of neuronal insulin signaling by trapping the INSR in endosomes [172]. A limitation of most published animal studies is that they measure basal INSR activation without stimulation by insulin, so the brain’s resistance to insulin remains uncertain. The little available evidence in insulin-injected animals suggests that INSR activation may still be triggered in animal models of AD, even following HFD-induced insulin resistance, similar to the treatment of advanced T2DM [115, 123, 166]. The possibility of a bidirectional relationship between AD and T2DM suggests that improving insulin signaling in the periphery or in the brain could break this cycle and be therapeutic in both diseases [115, 166, 173-175].

In summary, identifying BIR in HFD-fed animal models has proven more difficult than identifying peripheral insulin resistance. Discrepancies between studies stem from differences in the age of exposure to the HFD, as well as models used (AD or otherwise, as some of these models are prone to atherosclerosis), and endpoints assessed, with the lack of information on the exact diets used remaining the most constraining issue. Still, important gene-diet interactions have been uncovered, reinforcing the hypothesis of targeting brain insulin signaling in AD.

### 4.1b Toxin models

ICV injection of the diabetogenic toxin streptozotocin (ICV-STZ) is often described as a model of BIR or “sporadic AD”; however, this classification is problematic. On the one hand, some aspects of T2DM and AD are found with this approach. Indeed, ICV-STZ brains present with decreased brain glucose metabolism and impairment in the brain insulin-INSR system [176-179]. This model often displays a rise in neuroinflammatory markers, oxidative stress, reduction in cerebral energy metabolism, and functional changes including learning and memory deficits. While the method of action of STZ within the brain remains unclear, this toxin-based approach can increase Aβ and p-tau neuropathology levels in rodent models of humanized Aβ or tau [180-182]. In AD animal models, STZ-ICV injection was found to worsen neuroinflammation, cognition deficits, and neuropathology [180, 182-184]. However, BIR status has mostly been assessed by evidence of changes in the basal phosphorylation downstream INSR substrates without a direct insulin challenge [179, 182, 185]. In addition, ICV injection of STZ is known to also result in some STZ moving into the blood and inducing insulin deficiency, a model of T1DM, not T2DM, in the periphery [179] by selectively ablating the insulin-producing pancreatic beta cells. In sum, it is difficult with this model to determine whether the AD-relevant effects observed of STZ result from the induction of central (brain) or peripheral insulin resistance and how this model relates to T2DM or AD.

### 4.1c Genetic/inducible models

Other mouse models considered tools for investigating BIR include either genetic models of brain INSR deletion or the use of lentiviruses to knock-down or overexpress levels of the INSR in select regions. In 2000, the first brain-specific INSR knock-down mouse was generated [186]. Since then, many other models have been generated not only to investigate the role of INSR in the whole brain but also within select CNS-cell types, including endothelial cells located at the BBB [30, 187-189]. Most recently, these brain INSR-deficient models have been crossed with mouse models of AD to further interrogate the relationship between INSR and AD pathology [190].

One limitation of such approaches is that cells (e.g., neurons, astrocytes, microglia) from the whole brain are targeted. While cell-specific effects will be identified, effects due to sub-populations within these cell types will be lost. However, it is possible to selectively knock-out the INSR in selective cell populations, which was recently shown in a neuronal population. Martin *et al.* recently developed SeIRKO mice in which the INSR is selectively knocked-out in brain serotonergic neurons [33]. They showed that the anxiolytic effect of insulin is abolished in SeIRKO mice in response to intranasal (INL) insulin. More studies are needed to question the effect of insulin in other selective neuronal networks. Nevertheless, such a selective approach is important to evaluate the effect of insulin on the control of brain functions acutely, past the developmental stage as brain insulin signaling is involved in growth and development [64].

Another alternative approach to investigating the impact of BIR in various mouse models is to manipulate the brain INSR, using lentiviral or adenoviral techniques. This tool is particularly useful to study the impact of the INSR in various brain regions and specific cell types, in a variety of mouse models across the lifespan. Lentiviral-mediated knockdown can be used to reduce INSR levels locally [191]. Using this tool, the role of the INSR in the hypothalamus and hippocampus has been further elucidated in various physiological states [32, 191-194]. Alternatively, efforts to increase brain expression of a constitutively active INSR using an adeno-associated virus targeting neurons, show memory can be enhanced in aging [195]. Limitations to these approaches (both viral and knock-down) are that they do not typically completely eliminate the INSR and often do not target every cell type (i.e. microglia in particular are hard to target using adenoviral or lentiviral approaches). However, these approaches still prove to be useful in identifying cellular contributions for INSR signaling.

The selective INSR antagonist, S961 [196], is also useful for exploring the impact of acute insulin resistance on BIR under various conditions. For example, S961 has been used to identify the role of the INSR at the BBB on BIR and insulin BBB transport [41, 75, 197-199]. Additionally, S961 has been used to better understand the delivery of INL insulin to the brain [200]. S961 has also been used to induce BIR acutely to assess the impact on insulin BBB transport [201]. These tools highlight the pre-clinical models available for interrogating the role of BIR in AD.

### 4.2 Human post-mortem tissue

Post-mortem investigation of the human brain is another important and useful tool to study the presence of BIR in aging and its association with brain function, cognition, and dementia in particular [9]. Using human brain tissue to study BIR and signaling may provide insights into diseases that are uniquely human, including insights into mechanisms of disease [202]. Indeed, Talbot et al were the first to show in post-mortem human AD brain sections from the University of Pennsylvania and Rush University Medical Center cohorts, that BIR was present, defined by the ex vivo response to insulin stimulation, and that levels of pIRS1(S^616^, S^636/639^) negatively correlated with cognition [10] (Fig. 2). Nevertheless, the application of post-mortem investigations of BIR is still relatively limited because the post-mortem brain tissue cannot be easily acquired, especially of persons with and without AD and in whom detailed clinical data (e.g., cognitive data, dementia status, etc.) are available. Therefore, the ability to interrogate the relationship between BIR and genetic or lifestyle risk factors have largely been unexplored using this tool. Another caveat is that these individuals are usually at the later stages of disease progression so studying the development and progression of disease is difficult. Further, there are changes in brain structure, metabolism, and function that occur in the highly variable peri- and post-mortem interval which complicate the experiments and interpretation of results. Indeed, experiments suggest that a short post-mortem interval is important in order to successfully conduct ex vivo stimulation with insulin. Finally, the findings from human post-mortem studies may not be completely clinically translatable, which is a significant limitation in reaching the goal of prevention of cognitive decline in living persons with BIR.

### 4.3 In vivo neuroimaging

*In vivo* neuroimaging has the potential to provide measurements of a subset of the various physiological events characterizing BIR, with notable gaps and limitations. One of the most significant gaps in the neuroimaging-based assessment of BIR is the relative lack of techniques for assessing insulin transport across the BBB and binding to receptors in the brain. PET with a radiolabeled insulin ligand would appear to be ideal for this application because dynamic PET techniques, together with compartmental modeling techniques, have the ability to provide separate estimates of BBB exchange and receptor occupancy. However, to our knowledge, data from only one such radiolabeled insulin ligand has been published to date [203], and this data came from mice only. Lacking such insulin PET radioligands, PET radioligands that selectively bind to the insulin-like growth factor 1 (IGF-1) receptor, including radiolabeled IGF-1 itself, could be useful as proxy indicators of INSR binding due to the high level of cross-binding between these molecules and their respective receptors [204-206]. However, there is still relatively little work to date validating such IGF-1 ligands in living humans and post-mortem samples to understand the role they could play in the study of brain insulin action.

The individual biochemical events in the cascade of events that follow INSR binding—including IRS-1 phosphorylation, Akt activation, and downstream modulation of mTOR, GSK3, FOXO, and other pathways—are all difficult to measure directly using in vivo neuroimaging techniques. Glucose uptake into the cell, which is one consequence of these biochemical events in select cell populations, is however commonly measured using PET imaging with 18F-fluorodeoxyglucose (FDG), a glucose analog that is readily taken up by the cell in place of glucose. Because FDG PET imaging is clinically useful for the evaluation of a number of diseases, especially cancer but also AD, FDG PET is readily available at many research centers and now also in academic medical (clinical) centers, and it has been explored in conjunction with either peripheral or central insulin challenges as a proxy measure of BIR. In hyperinsulinemic clamp paradigms, circulating insulin levels are increased via continuous infusions of insulin; the amount of FDG taken up by the brain is compared between high and basal insulin conditions to assess how much additional glucose uptake is provoked by the increase in insulin [44, 47, 207]. Alternatively, somatostatin infusions allow assessment of the sensitivity of brain glucose uptake to reductions in steady-state circulating insulin levels [208]. Finally, naturalistic experiment designs seek to relate neuroimaging-based metabolic measures to modulations of circulating insulin levels that were caused not by the experimenter, but by the participant, via self-administration of insulin for diabetes treatment [209]. The presumed assumption that ties these studies to BIR is that changes in steady-state levels of peripheral insulin correspond tightly to changes in central levels. There is emerging evidence that brain production of insulin at the choroid plexus may follow a distinct regulation process than systemic insulin, driven by serotonin and not glucose signaling [39]. INL insulin challenges attempt to avoid this difficult assumption by administering insulin directly to the brain, with minimal spillover into the periphery [210, 211], but there are surprisingly few studies that relate INL insulin challenges to changes in FDG PET metabolism [212]. One study observed that individuals with AD or mild cognitive impairment (MCI) randomized to chronic INL insulin exhibited attenuated declines in FDG PET uptake over time compared to those randomized to placebo [212]. However, utilizing FDG PET solely as a marker for BIR proves difficult as 1) BIR is not simply tied to brain glucose metabolism and 2) insulin action has different effects on the vasculature (initiating vasodilation) versus the synapse (plasticity), which speaks to its role as a trophic factor or potentially, energy modulator. This highlights the need to improve our methods for assessing BIR or combine tools to further understand BIR in the context of AD.

Magnetic resonance spectroscopy (MRS) provides an alternative means for assessing brain glucose utilization responses to peripheral or central insulin challenges. Infusing a fuel substrate (such as glucose) labeled with 13-carbon (13C), together with the collection of 13C MRS measurements, allows for the simultaneous and dynamic measurement of both the 13C-labeled fuel and the by-products of its metabolism [213]. The glucose metabolic flux estimates resulting from such measurements could potentially be compared between differing insulin states, although human data using 13C MRS with 13C-labeled glucose and insulin manipulation has been limited [214]. Recent advances in 1-hydrogen (1H) MRS allow for measurement of the concentration of endogenous glucose in the brain with no need for a stable isotope infusion [215], although to date it also does not appear that comparisons of these measurements across differing insulin states have been made.

MRS also allows for the measurement of other molecular indicators of cell metabolism beyond glucose utilization. Classical 1-hydrogen (1H) MRS techniques allow for the measurement of neuronal function and integrity markers such as N-acetylaspartate (NAA) and choline-containing compounds (Cho), as well as the regulatory compound myo-inositol and the neurotransmitters glutamate, glutamine, and GABA (sometimes measured all together as a composite variable, Glx), and creatine (Cr) as an indicator of total energy use. Among these indicators, one study suggested that under hyperinsulinemic-euglycemic clamp conditions, the NAA/Cr and NAA/H2O ratios in frontal cortex, and the Glx/Cr and Glx/H2O ratios in frontal and temporal cortices, are elevated compared to basal conditions, while frontal Cho/Cr and temporal Cho/H2O and myo-inositol/H2O ratios are decreased relative to basal conditions [216]. In addition, 31-phosphorus (31P) MRS can provide dynamic measurements of endogenous brain levels of the energetic products adenosine triphosphate (ATP) and phosphocreatine (PCr), both of which appear to increase as soon as ten minutes after an acute INL insulin challenge [217]. However, it should be noted that the number of such MRS studies is very small, likely due to limited accessibility, and that further, the sample size in these studies is also very small, raising the possibility for biased results.

More common are studies that use dynamic magnetic resonance imaging (MRI) techniques to assess the downstream cerebrovascular consequences of altered neural and glial metabolic activity. Blood oxygenation level-dependent (BOLD) functional MRI (fMRI) dynamically measures local relative concentrations of oxygenated hemoglobin, which change with a complex time course in response to local changes in neural and glial metabolic demands [218]. BOLD fMRI accomplishes this using conventional MRI hardware, without the injections or ionizing radiation of FDG PET, and without stable isotope infusions. fMRI, used to image the brain’s default mode network (DMN) which is a network of regions that are more active at rest than during an effortful task, is disrupted in AD patients and those with increased AD risk [219]. Similarly, T2DM patients show differences in DMN functional connectivity compared with controls, and those differences are associated with measures of insulin resistance in selected brain regions [220]. These findings agree with FDG PET measures showing brain glucose hypometabolism in the same areas in T2DM patients [14, 221].

Because of its relative ease of use, changes in BOLD fMRI signal amplitude in response to glucose infusions and peripheral insulin infusion have been assessed. In one study, the BOLD signal amplitude in the resting state increased in a distributed cortical composite region of interest, as well as a hypothalamic region of interest, in response to a 2-minute steady-state 3 mg/kg of body weight infusion of glucose [222]. In another study, BOLD responses to finger tapping, simple reaction time, and four-choice reaction tasks differed in multiple brain locations during a hypoglycemic clamp compared to basal conditions [223]. In addition, changes in the resting-state inter-regional synchrony of the BOLD signal (the so-called “functional connectivity”) in response to INL insulin challenge have also been assessed. One of these studies suggested that functional connectivity between the right hippocampus and the medial prefrontal cortex lowers in response to a 160 IU acute dose of INL insulin [224]. Another study suggested that BOLD fMRI-based functional connectivity between the hippocampus and other regions, including the hypothalamus and prefrontal DMN regions, increased following an acute dose of INL insulin [35]. A third study suggested that the intrinsic local statistical characteristics of the BOLD signal in the hypothalamus and orbitofrontal cortex are modified by INL insulin [225]. Other studies have used a similar technique, perfusion MRI, to measure cerebral blood flow responses to an acute INL insulin challenge [225]. One such study suggested that cerebral blood flow in the bilateral amygdalae reduced significantly compared to basal conditions after an INL insulin challenge [226]. Another study suggested that right putamen cerebral blood flow increased in normal weight adults following INL insulin and that this response was blunted in overweight or obese adults [224]. Perfusion MRI differs from BOLD fMRI in that its measurements isolate cerebral blood flow, while BOLD measurements represent the culmination of cerebral blood flow, cerebral blood volume, and local oxygen metabolism. Note that while in vivo neuroimaging studies of brain responses to insulin challenges have largely focused on measurements from a single imaging modality, multi-modal studies that combine BOLD fMRI, perfusion MRI, and MRS appear to be on the way [227].

### 4.4 Cognitive response to insulin

Similar to imaging studies assessing the response to an external stimulus, such as insulin administration, an alternative approach assessing cognitive response can be used as a proxy for BIR. Insulin administration either via a hyperinsulinemic clamp [48] or via INL delivery [228-232] may enhance memory in selected populations [233]. Therefore, impaired cognitive responses to insulin administration in this setting may be a result of BIR, more specifically impaired insulin transport across the BBB resulting in insulin deficiency in the brain. Indeed, in individuals within populations known to have BIR, such as in ApoE4 individuals or those with AD or MCI, the response to INL insulin is impaired [212, 234-237]. Previous rodent studies have shown there are no differences in the brain distribution of intranasally-administered insulin, indicating the availability of insulin is not the issue with this administrative route [200]. Furthermore, there is a differential sensitivity to INL insulin between men and women [210, 238] and sex differences in brain insulin signaling have been noted [224]. Cognitive response to external insulin administration is an indirect method for assessing BIR and assumptions must be made. However, it is still a useful tool for elucidating relationships and tracking changes longitudinally. Nonetheless, challenges remain in the production of pharmaceutical formulations for the brain delivery of insulin which continue to make its’ use in research and translation into the clinic difficult [229].

### 4.5 Biofluid biomarkers

Biofluid-based indicators of the many physiological events involved in brain insulin processing would greatly improve the efficiency of learning about BIR in living human subjects because the current in vivo neuroimaging approaches described above are expensive, technically elaborate, and limited in tracking physiological processes over time. In addition, biofluid-based measures have the potential to assess physiological events that are currently not measurable by in vivo neuroimaging techniques. Markers of IRS-1, with various patterns of phosphorylation, derived from blood-based exosomes enriched for neuronal origin, are currently of intense interest. Phosphorylation of IRS-1 is one of the key biochemical steps leading from INSR binding to downstream metabolic effects. These exosomes are small extracellular vesicles that are released regularly by neurons, cross the BBB into circulation, and carry markers of their neuronal origin to enable their identification. A growing body of work has related the abundance of exosome-derived markers to standard AD-related markers and compared them between groups of differing clinical status [239-241]. CSF-based markers of insulin and insulin-related molecules are alternative biomarkers that avoid the complexities of BBB transport at the expense of a more risky, invasive procedure to obtain the biofluid. CSF-derived insulin concentration has been assessed in relation to AD clinical status and AD-related biomarkers [242, 243], to responses to dietary intervention [244], and to measures of structural brain aging [245]. Concentrations of various biomarkers related to insulin or to peripheral insulin resistance have also been assessed in CSF and blood of subjects from various cohorts, including those with AD (Table 2) [243, 246-248]. Assessment of blood or CSF levels of insulin does not yield consistent results but often have contradictory trends [242, 244, 247, 249-254]. In one study, higher insulin levels in the CSF were associated with worse global cognition and higher p-tau levels, particularly in women and in ApoE4 non-carriers [242]. Although results from studies are not in agreement, several report higher adiponectin and lower leptin levels in blood drawn from subjects with MCI or AD compared to controls (Table 2). More consistently, higher concentrations of IGF-1 [252, 255-257], IGF-2 [256, 258, 259], insulin-like growth factor binding protein (IGFBP)-1 [257, 258], IGFBP-2 [246, 256-262] and IGFBP-3,4,5 [252, 256, 257] have been associated with a diagnosis of AD (Table 2). Overall, despite considerable inter-study variability, biomarker studies depict a complex metabolic signature of AD, possibly including elevated concentrations of IGFs or IGFBPs in both CSF and blood [243, 246-248]. Despite these changes in CSF and blood biomarkers, omics-based signatures of brain insulin signaling from any biofluid, have been slow to develop [263].

**Table 2:** Summary of studies on insulin-related biomarkers in Alzheimer’s disease, using various assays in plasma, serum, cerebrospinal fluid, or brain homogenates.

Cohort (n)	Higher levels in	Body fluid or tissue	Summary of main results	Additional notes	Ref.
***Insulin***					
NCI/MCI,AD (21/19) NINCDS/ADRDA criteria	Ctrl	CSF	NCI > MCI/AD (.005)		*[251]*
	=	Serum	ns		
SCI/aMCI/AD (45/44/49)	AD (women)	CSF	aMCI < SCI < AD (.059) Women: Significantly different with MMSE	Women: higher levels associated with worsen global cognition APOE4 non carriers: correlation with CSF p-tau and t-tau	*[242]*
Non-elderly/elderly (116/96) with 65years-old as threshold, with MetS MoCA		Plasma		Non-elderly MetS: correlation with MoCA	*[344]*
Ctrl/sporadic AD (60/60) neuropsychological evaluation (DSM) + MRI	AD	Serum	Ctrl < AD (<.0001)		*[249]*
Ctrl/other dementia/stable MCI /AD (15/13/32/60)	=	Serum	ns		*[252]*
	=	CSF	ns		
Ctrl/AD (12/16) Braak staging	Ctrl	CSF	Ctrl > AD (.0009)		*[247]*
	Ctrl	Brain homogenate	B0-1 (Ctrl) > B6 (AD) (.05)		
Ctrl like or AD like (29/30)		CSF		CSF Ctrl like: inverse association between WMHs and insulin CSF levels (parieto-occipital region)	*[245]*
Ctrl/aMCI (20/29) with 4 weeks high-SFA/glucose (HIGH) diet or a low SFA/glucose (LOW)	AD	CSF	HIGH diet: Ctrl < MCI/AD LOW diet: Ctrl < MCI/AD		*[244]*
	AD	Plasma	HIGH diet: Ctrl = MCI/AD LOW diet: Ctrl < MCI/AD		
Ctrl/AD (24/21) Braak staging	Ctrl	FCx	B0-1 (Ctrl) > B2-3/B4-5/B6 (<.001)		*[345]*
Ctrl/AD (26/28) Braak staging	Ctrl	FCx, Hpc, Hyp	Ctrl > AD (Hyp .01; Hpc .002; ns FCx)		*[346]*
Ctrl/AD (16/27)	=	CSF	ns		*[253]*
Ctrl/mild AD/severe AD (25/14/11) Or Ctrl/homozE4/non-homozE4 (14/6/19)	AD	Plasma	Ctrl, mild AD < moderate/severe AD (.05) APOE4: normal, homoz ApoE4 < non homoz E4		*[250]*
	Ctrl	CSF	Ctrl > mild AD > moderate/severe AD (.05) APOE4 : ns		
Ctrl/AD (26/54)	AD	CSF	Ctrl < AD (.001)		*[254]*
	AD	Plasma (after OGTT/fasted)	Ctrl < AD (.001)(after OGTT); ns (fasted)		
***Leptin***					
Ctrl with neurological but not degenerative disease/AD (23/26)	=	Plasma	ns		*[347]*
*Cross-sec study 669 participants*		Plasma		Associated with cognitive impairment	*[348]*
Ctrl/MCI,AD (21/19) NINCDS/ADRDA criteria	=	CSF	ns		*[251]*
	Ctrl	Serum	Ctrl > MCI/AD (.0002)		
Ctrl/AD (25/30) Turkish MMSE, CDR, GDS	=	Plasma	ns		*[349]*
Ctrl/MCI with T2DM (63/61) MoCA	Ctrl	Plasma	Women: Ctrl > MCI	Associated with MoCA (higher levels associated with better cognition)	*[350]*
NCI/MCI/AD (21/8/13) NINCDS/ADRDA criteria	AD	CSF	MCI < Ctrl < AD (.05) Women > Men	↘ Leptin receptor in AD (CSF and hpc)	*[351]*
Ctrl/ AD (60/60) neuropsycho evaluation (DSM-IV) + MRI	Ctrl	Serum	Ctrl > AD (<.0001)		*[249]*
Ctrl/AD (12/16) Braak staging	=	CSF	ns		*[247]*
	AD	Brain homogenate	B0-1/B2-4 < AD (B6) (<.05)		
Ctrl/AD (37/41) NINCDS-ADRDA criteria	Ctrl	Plasma	Ctrl > AD		*[352]*
785 participants 111 incident dementia (89 AD)	Ctrl	Plasma	Ctrl > dementia/AD		*[353]*
***Adiponectin***					
Ctrl with neurological but not degenerative disease/AD (23/26)	=	Plasma	ns		*[347]*
535 non-demented elderly, with neuropsychological tests		Plasma		Women: inverse association with cognitive outcomes	*[354]*
Ctrl/sporadic AD (60/60) neuropsychological evaluation (DSM) + MRI	AD	Serum	Ctrl < AD (<.0001)		*[249]*
NCI/MCI/AD (51/65/41)	MCI/AD	Serum	NCI < MCI, AD (<.001)		*[355]*
NCI/MCI/AD (28/18/27) NINCDS-ADRD	MCI/AD	Plasma	NCI < MCI, AD		*[356]*
	MCI	CSF	NCI < MCI (ns AD)		
Ctrl/AD (37/41) NINCDS-ADRDA criteria	=	Plasma	ns		*[352]*
***Ghrelin***					
Ctrl/MCI,AD (21/19) NINCDS/ADRDA criteria	=	CSF	ns		*[251]*
	AD	Serum	Ctrl < MCI/AD (<.0001)		
NCI/MCI (30/22) neuropsychological tests	=	Serum	ns (total ghrelin)		*[357]*
	MCI	Serum	NCI < MCI (<.001) (acylated ghrelin)		
Ctrl/AD (12/16) Braak staging	Ctrl	CSF	Ctrl > AD (.005)		*[247]*
	=	Brain homogenate	ns		
***GIP***					
Cross sectional studies, 3001 older people MMSE and AQT		Serum (2h OGTT/fasted)		Correlation with MMSE (2h OGTT) (higher levels are associated with better cognition); ns (fasted)	*[358]*
Ctrl/MCI,AD (21/19) NINCDS/ADRDA criteria	MCI/AD	CSF	Ctrl < MCI/AD (.02)		*[251]*
	=	Serum	ns		
Ctrl/AD (12/16) Braak staging	=	CSF	ns		*[247]*
	mild NFT pathology	Brain homogenate	B0-1/6 < B3-4 (<.01)		
***GLP-1***					
Cross sectional studies, 3001 older people MMSE and AQT		Plasma (2h OGTT/fasted)		Correlation with MMSE (2h OGTT) (higher levels are associated with better cognition); ns (fasted)	*[358]*
Ctrl/MCI,AD (21/19) NINCDS/ADRDA criteria	=	CSF	ns		*[251]*
	MCI/AD	Serum	Ctrl < MCI/AD (<.0001)		
Ctrl/AD (12/16) Braak staging	Ctrl	CSF	Ctrl > AD (.012)		*[247]*
	Ctrl	Brain homogenate	B0-1 (Ctrl) > B6 (AD) (.05)		
***IGF-1***					
Ctrl/AD (36/ 40)		CSF		Correlation with CSF t-tau, p-tau	*[255]*
Ctrl/other dementia/stable MCI /AD (15/13/32/60)	=	CSF	ns		*[252]*
	AD	Serum	Ctrl < AD (.01)	Inverse correlation with CSF Aβ_42_	
Ctrl/AD (41 total)	AD	CSF	Ctrl < AD (.0001)		*[257]*
	AD	Serum	Ctrl < AD (.0001)		
Ctrl/AD (24/21) Braak staging	Ctrl	FCx	B0-1 (Ctrl) > B4-5/B6 (<.001)		*[345]*
Ctrl/AD (26/28) Braak staging	Ctrl	FCx, Hpc, Hyp	Ctrl > AD (Hyp .07; FCx .006; ns Hpc)		*[346]*
Ctrl/AD (10/10)	=	CSF	ns		*[256]*
	AD	Serum	Ctrl < AD (<.01)		
***IGF-2***					
Ctrl/other dementia/stable MCI/AD (20/15/13/32)	=	Serum	ns		*[258]*
	AD	CSF	Men: Con,MCI < AD	Correlation with t-tau and p-Tau	
Ctrl/AD (72/92)	AD	CSF	Ctrl < AD (.005)		*[259]*
Ctrl/AD (24/21) Braak staging	Ctrl	FCx	B0-1 (Ctrl) > B2-3/B4-5/B6 (<.05)		*[345]*
Ctrl/AD (26/28) Braak staging	Ctrl	FCx, Hpc, Hyp	Ctrl > AD (Hyp .01; Hpc .04; ns FCx)		*[346]*
Ctrl/AD (10/10)	AD	CSF	Ctrl < AD (<.01)		*[256]*
	AD	Serum	Ctrl < AD (<.01)		
***IGFBP-1***					
Ctrl/other dementia/stable MCI/AD (20/15/13/32)	=	CSF	ns		*[258]*
	=	serum	ns		
Ctrl/AD (41 total)	AD	CSF	Ctrl < AD (.0001)		*[257]*
	AD	Serum	Ctrl < AD (.0001)		
***IGFBP-2***					
1596 participants with 131 dementia cases including 98 AD cases	AD	Plasma		Associated with an increased risk of dementia and AD	*[260]*
NCI/MCI/AD (58/197/99) NINCDS/ADRDA criteria	=	CSF	ns		*[246]*
	MCI	Plasma	AD, NCI < MCI (<.0001)	Inverse correlation with episodic memory performance Amyloid-negative individuals (CSF Aβ_42_): Inverse correlation with hpc volume	
Ctrl/MCI/AD (45/134/66)				Correlation with t-tau	*[359]*
Ctrl/other dementia/stable MCI/AD (20/15/13/32)	=	Serum	ns		*[258]*
	AD (men)	CSF	Men: MCI < AD	Correlation with t-tau and p-Tau	
Ctrl/AD (72/92)	AD	CSF	Ctrl < AD (.005)		*[259]*
NCI/MCI/AD (211/149/331)	AD	Plasma		Associated with cognitive decline and AD diagnosis	*[262]*
Ctrl/AD from the AIBL cohort (754/207)	AD	Plasma	Ctrl < AD (<.0001)		*[261]*
Ctrl/AD (8/8) Braak staging	Ctrl	TCx	Ctrl > AD (.05)		*[360]*
Ctrl/AD (41 participants)	AD	CSF	Ctrl < AD (.0001)		*[257]*
	AD	Serum	Ctrl < AD (.0001)		
Ctrl/AD (10/10)	AD	CSF	Ctrl < AD (<.001)		*[256]*
***IGFBP-3***					
Ctrl/Other dementia/stableMCI /AD (15/13/32/60)	=	CSF	ns		*(Johansson et al. 2013)*
	sMCI/AD	Serum	Ctrl < sMCI, AD (.01)	Inverse correlation with CSF Aβ_42_	
Ctrl/AD (41 total)	AD	CSF	Ctrl < AD (.0001)		*[257]*
***IGFBP-4,5***					
Ctrl/AD (41 total)	AD	CSF	Ctrl < AD (.0001)		*[257]*
***IGFBP-6***					
Ctrl/AD (41 total)	AD	CSF	Ctrl < AD (.0001)		*[257]*
Ctrl/AD (10/10)	AD	CSF	Ctrl < AD (<.001)		*[256]*
***FGF21***					
569 participants (Ctrl/T2Dwo complications/T2Dw complications/AD (102/92/162/93/120)	=	Plasma	ns Ctrl versus AD (but higher in T2D versus AD) APOE4 status has no impact	Correlates with age as centenarian has the highest plasma level, and BMI	*[361]*
Ctrl/MCI (39/92) MoCA	MCI	Plasma	Ctrl < MCI (.004)		*[362]*
Nonelderly/elderly with 65 years-old as threshold, with MetS (116/96) MoCA		Plasma		Non-elderly MetS: Inverse correlation with MoCA (lower levels associated with better cognition)	*[344]*

Background colors: Red cells = Higher levels observed in MCI and/or AD; Purple cells = Brain tissue instead of CSF or blood *Abbreviations: (a)MCI, (amnestic) mild cognitive impairment; AD, Alzheimer’s disease; Aβ, β-amyloid; CDR, Clinical Dementia Rating; Ctrl/NCI, controle/no cognitive impairment; CSF, cerebrospinal fluid; DSM, Diagnostic and Statistical Manual of Mental Disorders; E4, apolipoprotein E4; ELISA, enzyme-linked immunosorbent assay; FCx, frontal cortex; FGF21, Fibroblast growth factor 21; GDS, Global Deterioration Scale; GIP, Glucose-dependent insulinotropic polypeptide; GLP-1, Glucagon-like peptide 1; Hpc, hippocampus; Hyp, hypothalamus; IGF, Insulin-like growth factor; IGFBP, Insulin-like growth factor binding protein; LIA, line immunoassay; MetS, metabolic syndrome; MMSE, Mini-Mental State Examination; MoCA, Montreal Cognitive Assessment; MRI, magnetic resonance imaging; NFT, neurofibrillary tangles; NINCDS-ADRD, National Institute of Neurological and Communicative Diseases and Stroke/Alzheimer's Disease and Related Disorders Association; OGTT, Oral glucose tolerance test; RIA, radioimmunoassay; SCI, subjective cognitive decline; SFA, saturated fatty acids; T2D, type 2 diabetes; TCx, temporal cortex; WMHs, white matter hyperintensities.*

## 5. Correlation of BIR with AD hallmarks

### 5.1 AD Hallmarks

The Jack et al. model of the progress of pathophysiologic processes in AD was first published in 2010 and since expanded upon, describes a hypothetical model of the temporal sequence of AD biomarkers intended to be a framework for in vivo staging of AD [264, 265]. Measures of Aβ deposition and neurodegeneration were the primary markers for AD, with Aβ deposition occurring earlier, then followed by tau-mediated dysfunction, and later by the development of memory impairment. This model was quickly revised in 2012 to include advancements in amyloid imaging, MRI, and FDG PET. Most notably, the current definition of AD is the AT(N) framework, which states that a definitive diagnosis of AD must include Aβ, tau, and some aspect of neurodegeneration at autopsy [266]. A range in cognitive impairment is still last to follow these biomarkers. Since then, many have proposed modifications to the model, including adding a vascular component given most AD is accompanied by vascular abnormalities. Such an addition is pertinent to T2DM and insulin resistance, among other vascular risk factors. While we recognize many other factors may play a role in the development of AD, including neuroinflammation, oxidative stress, and changes in miRNA, we propose BIR also fits into this framework. We do not know where BIR falls in the development of AD. However, recent efforts to identify where in the course of disease progression BIR begins to develop have begun to be explored, and we suspect BIR may develop before the manifestation of cognitive impairment [41]. Further, we do not yet know whether BIR is a cause or a consequence of AD neuropathology, or a more independent factor in brain aging which relates to cognitive impairment. Unfortunately, the molecular pathways involved in BIR and the interactions with AD-related pathology are largely unknown but could provide valuable mechanistic insights that enhance our understanding of the disease process. In efforts to further define this relationship, investigators have begun to interrogate the development of BIR as it relates to AD pathology in both rodents and humans. We discuss in this section how the tools for interrogating BIR described above can aid in correlating directly to hallmarks of AD defined by the Jack et al. model.

### 5.2 Rodent models

As described above, animal models can be used to directly probe BIR in vivo, in association with AD biomarkers. Rodent models are particularly useful on the one hand to generate a phenotype of obesity and diabetes, and on the other hand to generate brain AD-like neuropathology, to study the causal interactions between both diseases. Mouse models of AD often display glucose impairment and insulin intolerance, exacerbated with HFD [149]. However, the correlation between BIR and AD pathology has not largely been assessed. BBB insulin interactions are impaired in the aged SAMP8 model of AD, after the presence of increased Aβ, compared to young controls [267]. INSRα-B protein levels at the BBB are significantly reduced in another AD mouse model, the 3xTg-AD mice, at 18 months of age compared WT controls, with a significant linear trend from 6 to 18 months of age [41]. Additionally, in the 3xTg-AD mouse model, insulin-induced activation of the INSR at the BBB is blunted, beginning at 6 months of age [41]. In female 3x-Tg-AD mice, pathological development of Aβ deposits, p-tau, and cognitive deficits typically appear by 6 months of age [268]. These data support the link between BIR and AD hallmarks but also suggest that BBB insulin resistance may correlate with AD hallmarks as well.

Other studies have tried to investigate the relationship of BIR with AD pathology. The A7-Tg AD mouse model deposits Aβ at approximately 12 months of age [143]. At 3 months of age, prior to changes in the insoluble levels of Aβ, an experiment placed mice on a HFD. After just 2 months on this diet, the cerebral cortex INSR response to peripherally administered insulin was blunted, but insulin BBB transport was also decreased. At 9 months of age, insoluble Aβ was significantly increased in the cerebral cortex and attributed to the HFD. These data suggest impairments in insulin BBB transport may impact brain insulin signaling and that these effects precede AD pathology. One caveat to many of the mouse models of AD is they do not typically follow the natural progression of the disease, resulting in accelerated AD neuropathology. Indeed, pre-clinical studies may not translate completely to human responses.

### 5.3 Post-mortem human brain tissue

Complementary investigations have been performed in post-mortem human brain tissue. In an earlier study of the post-mortem brain cortex of 17 older patients with AD, the densities of INSR were significantly increased, compared with age-matched controls [269]. Correlations to the levels of AD hallmarks in the same samples were not performed. A larger study using post-mortem examinations of the brain in AD cases demonstrated that the hippocampal formation and cerebellar cortex of patients with AD compared to age-matched controls exhibited markedly reduced responses to insulin signaling in IRS-1/IRS-2 and PI3K pathways, seemingly irrespective of T2DM status and total INSR protein level [10]. In the same study, biomarkers of brain insulin signaling, in particular IRS-1, were positively associated with increased levels of oligomeric Aβ plaques. Further, phosphorylated IRS-1 was negatively associated with cognition, including episodic memory, the cognitive domain typically affected earliest in the clinical expression of AD [10]. Similarly, leveraging post-mortem human brain tissue, evidence of BIR was also found in patients with amnestic mild cognitive impairment and early-onset AD in Down syndrome [270, 271]. Hyperphosphorylation of mTOR positively correlated with Aβ [270]. INSR and IRS1 levels positively correlated with the synapse markers, syntaxin, and PSD95, and this impairment in INSR signaling occurred early in Down syndrome before the development of AD pathology [271]. In a community-based cohort study of older persons with and without T2DM who came to an autopsy, brain Akt phosphorylation, a downstream event in the insulin signaling pathway, was associated with the level of AD neuropathology and lower cognitive function [202].

In post-mortem human brain microvessels, significant inverse correlations exist between INSRα-B levels and neuritic plaques, but not with parenchymal Aβ_40_ or Aβ_42_ [41]. Furthermore, there was a positive association between INSRβ and total soluble tau and an inverse relationship with neurofibrillary tangles. These data continue to support a relation not only between BIR and AD pathology but also between BBB insulin resistance and AD.

### 5.4 Biomarkers

Several studies have used brain insulin-related measurements from living humans to suggest that these biomarkers differ between cognitively normal older adults and those clinically diagnosed with AD, AD prodromal syndromes, and different neurodegenerative diseases [243, 272]. In addition, some of these studies have further assessed associations between fluid biomarkers of brain insulin signaling and biomarkers associated with AD [240]. Two studies have assessed whether chronic INL insulin administration modified AD biomarkers, with one showing null findings [212] and the other suggesting that the intervention modified CSF tau-P181/Aβ_42_ ratio [273]. Observational studies have suggested that various brain insulin-related measures extracted from blood or CSF are associated with MRI-based measures of neurodegeneration [240, 246, 274, 275] although the degree to which the spatial pattern of neurodegeneration is suggestive of AD is mixed. Indeed, some authors explicitly suggest that the neurodegeneration associated with brain insulin markers may be non-AD-related because findings were only evident in amyloid-negative individuals [246]. There have been two reports that brain insulin markers are associated with CSF tau burden [242, 274], and seemingly no reports that the brain insulin measures are associated with amyloid burden specifically (except for the aforementioned tau-to-amyloid ratio data in [273]). Yet, methodological heterogeneity in the literature on correlations between brain insulin markers and AD markers is high, with differing papers using CSF or blood to quantify insulin, a variety of insulin-related hormones or proteins, and exosome-derived molecules. Populations under study were also heterogeneous with respect to age, clinical status, and AD biomarker status. In addition, seemingly absent from this literature are neuroimaging-based measures of brain insulin, amyloid, and tau.

## 6. CNS cell specificity and regional localization

Thus far, research surrounding BIR is predominantly approached from a whole brain or individual region point of view, although advancements in interrogating the impact of insulin signaling in sub-cellular populations are evident [33]. A central question to consider is whether the brain is considered wholly an insulin-sensitive organ or whether insulin sensitivity varies across types of brain cells (e.g., neurons, astrocytes, endothelial cells) and regions, dependent on the age, disease, or stage of disease, and other factors (e.g., exposures). The very definition of BIR could also differ for each type of cell or region in the brain. However, with the recent advances in single-cell data and omics-driven data, researchers are more aware that specific cell populations may be drivers or initiators of a given pathology.

### 6.1 Neural cell types

As briefly introduced above in Section 4.1c, recent work on the impact of CNS cell-specific characterization of the INSR has been explored in the context of BIR. In mice that lacked the neural INSR, cognition is preserved, but there was also no change in metabolic parameters either including body weight, food intake, and adiposity [186]. This model has a lifelong loss of INSR signaling, so compensation from other signaling pathways is likely present. Additionally, this model was driven by the CaMKII promoter, which is expressed in an array of CNS cell types in the mouse including neurons, oligodendrocytes, and macrophages and is not as specific to neurons in humans as we now know [106].

Lentiviruses targeting the INSR are packaged into the human cytomegalovirus (CMV), which primarily targets neurons [276]. This knock-down model, when delivered to the hippocampus, displays impaired cognition compared to mice treated with the control lentivirus [194]. On the other hand, over-expressing the hippocampal INSR using a constitutively active INSR in aged rats enhanced memory recall [277] and neuronal glucose metabolism [195]. Adeno-associated virus 9 (AAV9) vectors are designed to primarily target neurons using the neuron-specific human synapsin-1 promoter. Neuronal-specific knock-down of the INSR using the same synapsin-1 Cre driver does not affect the mortality of the Tg2576 AD mouse model, unlike the loss of the IGF-1R [278]. The impact of knocking-down or increasing the INSR solely in the hippocampus in the presence of AD pathology remains to be determined.

Genetically targeted knock-down of the INSR can also be useful to pinpoint cell types involved. Using electrophysiological and behavioral approaches, it was recently shown that serotoninergic neurons of the raphe nuclei can display insulin resistance in HFD-fed animals [33]. Neuronal INSR signaling is not the only CNS cell type investigated in its cognitive role. An astrocyte-specific knock-down of the INSR, both constitutive and inducible, has also been generated. Genetic knock-down of the astrocytic INSR in adult mice displayed no change in cognition compared to controls [190]. However, when this genetic model was crossed with a mouse model of AD, loss of the astrocytic INSR resulted in further impaired cognition and enhanced amyloid plaque deposition, indicating a protective role of the astrocytic INSR against AD pathology and cognitive decline. Additionally, the astrocytic INSR is involved in neurovascular coupling, with decreased brain perfusion occurring in older mice [279].

### 6.2 BBB cell types

In order to better define the cellular localization of BIR, one has to consider the prominent role of the BBB located at the interface between the blood and the brain. Blood flow in the brain is directed through a vast network of capillaries where the BBB plays a key role in the exchange of nutrients and physiological information between the periphery and the brain. The adult human brain has roughly 400 miles of blood vessels, making it the most vascularized organ in the body [280]. This is a large surface through which the periphery interacts with the CNS, where each brain cell is irrigated by a capillary [281]. Transporters and receptors expressed by the BBB can regulate not only the transport of blood-derived compounds but also their downstream signaling.

Since most insulin is produced by the pancreas, then released into circulation, it is logical to assume that most of it will interact first with the BBB before having any impact on cells located deeper within the brain. Emerging work suggests that some insulin gets to the brain by crossing the relatively permeable choroid plexus through the blood-CSF barrier (BCSFB) [282, 283]. However, the surface area of the BBB is manifold larger than the BCSFB [280, 284] and the majority of nutrient exchange occurs at the level of the capillaries. Although considered a barrier due to its specific capacity to block the entry of small polar molecules, the BBB is actually formed by a complex nexus of cells, such as endothelial cells, pericytes, astrocytes, microglia, and neurons, forming a neurovascular unit (NVU) [285-288]. Whereas a neuron-centric perspective has prevailed in the past [65, 69, 289], the role of the BBB and the NVU in brain insulin response is now being increasingly recognized [30, 41, 42, 60, 74, 290]. Since increases in INSR phosphorylation in brain endothelial cells following intracarotid insulin administration is particularly strong compared to the parenchyma [41], it is important to consider the role of the BBB in the response. Previous work has also demonstrated that removing INSR from astrocytes or neurons impacts insulin signaling as well [90, 291-293]. These cells in culture respond to low concentrations of insulin, likely similar to those in the brain ISF [294, 295]. In summary, although a majority of INSR in the brain is found in the NVU, a significant portion is present in other cells within the parenchyma, where they can interact with low levels of insulin.

The regulation of INSR expression or post-transcriptional events has also been proposed as mechanisms leading to insulin resistance. Studies in the liver indicate that the amount of biologically active INSR at the cytoplasmic membrane can be reduced by its cleavage by the β-site amyloid precursor protein cleaving enzyme 1 (BACE1), particularly in T2DM [296]. The use of BACE1 inhibitors to enhance insulin signaling has thus been proposed [296-298]. Within the AD BBB, a strong association between INSRα-B, BACE1, and APPβ-CTF was found in microvessels, suggesting that INSRα-B is reduced along with an increase in BACE1 activity [41]. This suggests that BIR at the BBB may involve BACE1 cleavage of INSR, as was recently shown in the liver and plasma [296-298].

### 6.3 Regional involvement in BIR

Studies investigating the regional impact of the INSR on memory and cognition in rodents have only recently begun to be explored. Targeted knock-down of the INSR in the hippocampus impaired memory [194] while knock-down in the hypothalamus may have an indirect effect on memory through changes in whole-body metabolism [193]. These data suggest that while the INSR itself may contribute to cognition within the hippocampus, the INSR in other regions, including the hypothalamus, may indirectly be involved in mediating cognition through changes in whole-body metabolism. However, whether some regions are more vulnerable to BIR and why has not been investigated. To do this, a vast descriptive study investigating the development of BIR across brain regions as AD progresses would need to be undertaken. This could be accomplished using any of the tools described in Section 4 but would benefit most by investigating the in situ regional response to insulin stimulation.

Two separate lines of neuroimaging work have addressed the degree that brain responses to insulin, defects in brain responses to insulin, and brain byproducts of such defects, may be region-specific. First, the set of studies assessing neuroimaging responses to peripheral or INL insulin challenges naturally sought to assess whether those responses are region-specific. Among these studies, FDG PET responses to acute insulin challenges were fairly global [47, 207, 208] or null [44]. Functional and perfusion MRI responses to insulin-related challenges have been inconsistent across studies but have featured the prefrontal cortex, hypothalamus, cerebellum, amygdala, hippocampus, and occipital cortex [222, 223, 225, 226, 299]. Second, a group of studies sought to determine whether chronic brain insulin exposures or biomarkers of brain insulin signaling are associated with localized measures of brain volume from structural MRI, to investigate whether greater levels of brain insulin signaling may have long-term beneficial effects for brain tissue survival in specific regions. The bilateral parietal-occipital junction, middle temporal gyri, temporal lobe generally, and hippocampi were mentioned as regions whose volumes correlated with various brain insulin measures [239, 240, 246]; chronic INL insulin was associated with greater preservation of tissue volumes in a broad range of cortical regions including several frontal, temporal, and parietal regions that have been implicated in the pathological progression of AD [273]. Interestingly, in one study the spatial pattern of preserved brain tissue associated with elevated measures of brain insulin signaling was similar to the spatial pattern of IRS-1 expression in a standard brain atlas, consistent with the beneficial effects of brain insulin signaling localized to sites of IRS-1 action.

### 6.4 Discovery-driven omics approaches

Applying omics approaches for studying BIR phenomenon directly in humans is not straightforward due to the complexity of defining the phenotype. Many of the high-throughput transcriptomics and proteomics studies invariably used mouse or rat models. More recently however, metabolomics have been utilized to understand the global metabolomic changes occurring in the brains and serum of patients with AD, followed up by validation with the APP/PS1 mouse model of AD, as a means to identify new therapeutic targets [300]. The question about BIR and the effect of insulin can be posed in two corresponding ways. One way is to evaluate the differences in gene expression between control and BIR mouse models. An alternative approach is to evaluate the response to insulin treatment, most often via INL delivery. Although these are different questions, they revolve around the same phenomenon of BIR and its rescue with INL insulin. Thus, presumably omics investigations could point to important mediators.

One of the earlier studies investigated the effect of INL insulin using an aged F344 rat model [301]. Although 3-months of INL insulin treatment did not result in significant memory improvement, the effect was noted by a decrease of insulin binding (quantified using a ^125^I-labled insulin assay) in the thalamus but not the hippocampus. However, according to microarray data, the hippocampal gene expression pattern was altered both by aging and most importantly by INL insulin treatment. Out of the pathways upregulated by INL insulin, of particular interest are anti-inflammatory and synaptic stabilization. In addition, there is also an observation of antiproliferative and tumor suppressor pathways. This counterintuitive observation can be explained by the fact that over the long-term treatment, the hippocampus became less responsive to insulin, potentially as part of negative feedback, which is often not exhibited in the clinical setting [232, 235, 302].

An alternative animal model of BIR is the SAMP8 mouse that is prone to accelerated aging [303]. The improvements in memory of SAMP8 mice upon short-term or repeated INL insulin administration were statistically significant [304]. In the SAMP8 mouse model of AD, a single INL insulin treatment altered the expression of over 300 genes within the hippocampus, just four hours following administration [305]. The majority of these genes were involved in pathways such as T-cell receptor signaling, cell adhesion molecules, cytokine-cytokine receptor interactions, and chemokine signaling. Noteworthy, the pathways related to inflammation were altered similar to the study described above using aged rats, rather than direct changes in the insulin signaling pathway [301].

To answer the complementary question of what gene expression patterns are associated with BIR to begin with, omics approaches can be applied directly to the characterization of the hippocampus or other areas of interest directly to BIR mouse models. For example, transcriptomics analysis of the cortex of the HFD mouse models [306] showed an increased expression of genes related to immune response, such as Trem2 and Tyrobp, known to be part of the innate immune system. The down-regulated genes are related to neuron projections and synaptic transmission. Although, from this study, it isn’t clear if the impact on the neurons is the consequence of inflammation or an independent process. Yet, the theme of increased metabolism-related inflammation involving BIR is consistent with other models.

A similar pattern was observed in SAMP8 mice when compared to cognitively normal SAMR1 mice [307]. Further proteomic analysis in the same study of the hippocampus revealed a number of affected pathways. Specifically, the majority of the differentially expressed proteins are involved in the cytoskeleton and cell motility regulation. Cholinergic components involving the receptors and esterase enzymes showed a downward trend in the case of accelerated aging. However, the inflammatory cytokines were up in SAMP8 mice, consistently with the observations in other models and study designs.

Perhaps the primary reason for the hippocampus getting much attention is because of its role in AD. However, other brain components such as the BBB [30, 305] and choroid plexus, at the junction of the BCSFB [39, 308], have also been crucially implicated in insulin function in the brain, as discussed. Future discovery-type omics studies with a broader scope encompassing the brain components should be informative for understanding the roles of different regions and structures within the brain.

Several follow-up questions on BIR model experiments remain, and include the identification of the specific cell types, and which brain region(s) is playing the key role. One approach to address these questions is through deploying one of the emerging sequencing techniques that is based on isolation and sequencing of the RNA contents of the individual cell nuclei [309, 310]. Alternatively, one can make a hypothesis on the role of a particular cell type and perform an experiment on isolated primary cell culture.

Investigations of the primary murine astrocytes confirmed that to completely blunt the effect of insulin, knock-out of both INSR and IGF1R is required [311]. Regardless of the mediating receptor, the RNA-seq data from this study indicates that insulin stimulation, rather than IGF-1 stimulation, triggers the suppression of autophagy genes. At the same time, stimulation caused upregulation of the ribosomal and heat shock protein, highlighting the regulation of proteostasis. A broader effect also involved cholesterol synthesis and mitochondrial homeostasis.

An informative study using immortalized preadipocytes isolated from genetically engineered mice dissected the effect of different INSR domains on the signaling [312]. Although the source of the cell isn’t from the brain, the connection can be drawn based on the relevance of the insulin signaling between the brain and adipose tissues. The study, using a skillful combination of genetic engineering and liquid chromatography with tandem mass spectrometry (LC-MS/MS) phosphoproteomics, has demonstrated an alternative INSR signaling pathway, that is independent of the ligand and tyrosine-kinase activity, abbreviated as LYK-I. These new signaling actions of the INSR include changes in the phosphorylation of proteins involved in the regulation of extracellular matrix organization, cell cycle, and immune-related pathways. LYK-I of the INSR increases the cellular sensitivity to apoptosis. Whether changes in INSR protein expression in pathophysiological conditions, such as aging or AD, drive the LYK-I response remains to be answered.

Beyond just the study of the overall gene expression changes between control and BIR animal models or in response to INL insulin, omics techniques can play a further role in elucidating the mechanisms of pathology and treatment. As mentioned, single nucleus RNA sequencing (snRNA-seq) will be a valuable tool that can identify a specific cell type involved in pathology and/or response to INL insulin. Once the cell type is determined, the omics techniques aimed at functional interrogation of the signaling pathways or individual proteins will be of particular interest. Ideally, such studies should be conducted on primary cell cultures as this would allow more accurate control of the experiments. Similar to the aforementioned study [312], insulin treatment in combination with LC-MS/MS proteomics should reveal the exact mechanistic differences and effects associated with pathology and treatment. Isolation of individual proteins, such as INSR or IRS-1/2 adaptors, combined with LC-MS/MS would provide an in-depth characterization of the post-translational modification and binding partners dynamics. Finally, integrated multi-post-translational modification characterization approaches have been demonstrated to provide valuable information on the functional state of the proteome [313-315]. The most impactful applications are those designed to focus on a very specific question to avoid the complexity of interpretation with multi-omics studies.

## 7. Remaining Questions/Concepts

Many questions about insulin resistance in the brain remain. Given the current state of knowledge, we reiterate our definition of BIR: a complex condition that encompasses one or more causes for an inadequate response of the brain and cerebral vasculature INSR to insulin, including as it may relate to CNS insulin availability, CNS INSR and isoform expression, and/or downstream signaling events of the INSR in the brain. So far, data suggest that BIR correlates with cognitive impairment [10]. Why and how BIR arises remains to be elucidated and investigated. And, importantly, the functional and clinical consequences of BIR, including in aging and AD, continue to be explored.

Despite the clear link between BIR and AD, there are potential confounding factors that likely influence this relationship. Indeed, there are many factors that can lead to peripheral insulin resistance and cognitive decline including genetic predisposition, lifestyle factors, and co-morbidities. Importantly, ApoE4, which is a genetic risk factor for AD [317] and for heart disease and stroke [318, 319], has been shown to interact with BIR in the impairment of cognition [316]. Further, lifestyle factors such as exercise and diet can also impact BIR and are involved in cognitive decline, including independently from AD [320]. In a clinical study just last year, it was shown that a sedentary lifestyle, resulting in obesity, leads to BIR, and that exercise could reverse this [321]. Additionally, smoking can cause peripheral insulin resistance [322] and faster cognitive decline [323]; however, the impact on BIR is largely unknown. Other co-morbidities including vascular and sleep disorders that are associated with peripheral insulin resistance and have also been found to worsen cognitive decline. For example, white matter hyperintensities on brain MRI scans are often attributed to underlying cerebral small vessel disease, are known to be associated with cognitive impairment, and found to be increased in persons with peripheral insulin resistance found on brain PET scans [324]. Furthermore, INI reduces white matter hyperintensity progression and improves cognition [231]. Sleep dysfunction is well documented to negatively impact insulin sensitivity and metabolism [325-327]. Sleep dysfunction is also associated with AD, leading to Aβ accumulation [328-331], but the relationship with BIR is unknown. These and other confounding factors highlight the complexity of the BIR-AD relationship. Furthering our understanding on the mechanisms between BIR (and not just peripheral insulin resistance), cognitive decline, and AD pathology will help us more fully identify these interactions.

One important knowledge gap regarding the association between BIR and dementia attributed to AD or otherwise involves resilience, which can be broadly defined as a successful adaptation, adjustment, and compensation process that occurs despite exposure to significant adverse factors including pathology [332]. Indeed, T2DM and insulin resistance can substantially increase the risk of dementia. Yet, there are older people who have T2DM or insulin resistance but do not develop cognitive impairment during their lifetime. In line with this clinical observation, a pre-clinical study using brain/neuron-specific insulin receptor knockout mice (NIRKO) found that rodents with BIR exhibited intact short- and long-term learning and memory despite having significantly increased levels of p-tau protein, a hallmark of neurodegenerative diseases [293]. While in an animal model, this finding suggests that BIR alone may not be sufficient for the development of dementia, and cognitive resilience may be at play in engaging compensatory mechanisms. Although the exact mechanisms are still waiting to be elucidated, emerging studies show that both genetic factors (e.g., a resilience gene along the bile acid metabolism pathway) and non-genetic factors (e.g., physical and cognitive activities and social connectedness, among others) may contribute to cognitive resilience [333-338]. Conversely, it should also be noted that not all people with dementia develop BIR. The underlying mechanisms also largely remain unknown, but may critically involve modifiable lifestyle behaviors such as exercise and a healthy diet, which have been associated with brain insulin sensitivity in animal studies [339, 340]. For example, by combining INL administration of insulin with functional MRI of the brain, a recently published clinical trial further demonstrated that an 8-week exercise intervention could restore brain insulin sensitivity in sedentary adults who are overweight or obese [321]. Further, studies on subjects who have undergone bariatric surgery might also provide some answers. Besides improving insulin resistance, Roux-en-Y gastric bypass was recently shown to decrease cerebral glucose metabolic rate and improve cognition [341]. This finding suggests that BIR is not a fixed trait but is modifiable and potentially a treatable state.

Improving our tools to study BIR in living humans will aid our understanding of how BIR relates to AD. Generating a PET ligand that is sensitive enough to detect changes in insulin BBB transport and visualize regional access to insulin in vivo would be one advancement. Further enhancing the sensitivity of some of the current biomarkers, present in both CSF and blood, including extracellular vesicles displaying markers for BIR, would also aid in tracking the progression of BIR alongside other pathological hallmarks of AD. The advancement of these tools would allow us to identify whether ameliorating BIR has any impact on the clinical progression of AD or impairments in cognition, which has been shown repeatedly in pre-clinical studies. Moreover, using a combination of tools, mentioned here and novel tools being developed, offer the opportunity to understand at a more granular level what processes are involved in BIR. And, studies are needed that use different approaches, from cell culture to animal models and human studies, including human studies of diverse populations that are clinic- and community-based, and observational and interventional (clinical trials). Given the large and growing public health challenge of dementia attributed to AD, research needs to focus on potentially modifiable factors such as BIR. While research has come a long way in this regard, much work remains to identify plausible therapeutic targets in BIR, for the treatment and prevention of cognitive decline and dementia.
